# Advances in metabolomics of biomarkers for ischemic stroke: From bench to clinic

**DOI:** 10.4103/NRR.NRR-D-24-01384

**Published:** 2025-07-05

**Authors:** Jiaxin Sun, Chenxin Xiao, Jingyuan Zhang, Feng Lin, Yue Xu, Yanyu Li, Lei Zhang, Wenli Chen

**Affiliations:** 1 Department of Cerebrovascular Disease, The Fifth Affiliated Hospital, Sun Yat-sen University, Zhuhai, Guangdong Province, China; 2 School of Pharmacy, Macau University of Science and Technology, Macao Special Administrative Region, China; 3 Department of Pharmacy, The Fifth Affiliated Hospital, Sun Yat-sen University, Zhuhai, Guangdong Province, China; 4 Guangdong-Hong Kong-Macao University Joint Laboratory of Interventional Medicine, the Fifth Affiliated Hospital, Sun Yat-sen University, Zhuhai, Guangdong Province, China

**Keywords:** biomarker, energy metabolism, excitotoxicity, glutamic acid, glycerol, ischemic stroke, metabolomics, oxidative stress, patient, rodent

## Abstract

Ischemic stroke, a neurological impairment caused by cerebral vascular occlusion, accounts for 87% of the cases of stroke. Recent studies have shown that changes in the abundance of metabolites can directly reveal the cellular phenotypes and identify the clinical implications of stroke diagnosis and therapy. However, systematic research to clarify the relationship between biomarkers and the mechanisms of ischemic stroke remains limited. In this study, we reviewed articles on ischemic stroke metabolites from 2005 to 2024, identified metabolites showing significant changes, and constructed a metabolite database based on the findings from 128 studies. The database included 125 differential metabolites detected in a middle cerebral artery occlusion mouse model, 246 detected in an middle cerebral artery occlusion rat model, and 764 identified in ischemic stroke patient samples. Differential metabolites from various samples were then screened and classified into positive and negative categories based on their correlation with stroke prognoses. Based on this analysis, three positive metabolites and two negative metabolites were identified. Glutamic acid, glycerol, and 1-octadecanoyl-sn-glycero-3-phosphocholine (LysoPC(18:0)) were further recognized as potential biomarkers. Imbalances in metabolic pathways such as alanine, aspartate, and glutamate metabolism as well as the citrate cycle (tricarboxylic acid cycle) were analyzed. These imbalances may influence the pathogenesis of ischemic stroke by altering biological processes such as excitotoxicity, oxidative stress, inflammation, and energy metabolism. The identification and analysis of these potential biomarkers may provide valuable targets and strategies for prediction, diagnosis, and prognostic assessment of ischemic stroke.

## Introduction

Stroke is characterized by high rates of incidence, disability, mortality, and recurrence and an extremely high financial burden. According to projections, by 2030, the prevalence of stroke is expected to have increased by 20.5% from that in 2012 (Ovbiagele et al., 2013). Ischemic stroke (IS), which accounts for 87% of all cases of stroke, refers to the irreversible infarction caused by interruption or reduction of cerebral blood flow (Saini et al., 2021). Due to the complexity of pathological processes underlying IS and a short therapeutic window, expedited diagnosis and management of IS are of critical importance.

Recent studies have shown that metabolite alterations play major roles in the pathological mechanisms underlying IS, such as excitotoxicity, oxidative stress, inflammation, and energy metabolism, thereby providing new insights into the promising role of metabolic biomarkers with potential predictive, diagnostic, and prognostic value (Chen, 2022; DeLong et al., 2022; Neves et al., 2023; Yadav et al., 2024). Although a few metabolites, such as homocysteine, have been identified as risk factors for cardiovascular and cerebrovascular diseases and are routinely used as clinical diagnostic biomarkers, some limitations remain to be addressed, including an insufficiently systematic approach and inadequate guidance for prediction, diagnosis, and prognostic assessment. Additionally, current metabolomics techniques have been predominantly applied to individual animal models or a limited number of clinical samples; therefore, the broad applicability of the conclusions drawn from such studies are yet to be thoroughly established.

Metabolites are molecules smaller than 1.5 kDa, such as amino acids, lipids, and nucleotides. With advancements in multi-omics techniques and bioinformatics, a growing body of metabolomics studies has shed light on the potential role of metabolites. As a field of study covering metabolites, metabolomics can serve as a prospective approach to directly reveal cellular phenotypes and characterize the influence of the upstream input of the environment and the downstream output of the genome (Li et al., 2023). Moreover, it can facilitate the development of precision medicine by analyzing metabolic phenotypes of each patient. Metabolomics offers distinct advantages over proteomics and transcriptomics for identifying biomarkers of IS, since it can detect small-molecule metabolites that readily penetrate the blood-brain barrier (Ke et al., 2022). Furthermore, untargeted metabolomics, which profiles a broad spectrum of metabolites, can provide a comprehensive picture of the intricate complexity inherent in IS (Ke et al., 2022). Consequently, metabolomics is particularly well-suited to addressing the challenges posed by the blood-brain barrier and disease heterogeneity in the context of IS. However, only 1/7 known circulating metabolites have been quantified (Wishart et al., 2022), and few studies to date have systematically illuminated the functions of biomarkers for IS. Moreover, summaries of the potential relationship between biomarkers and mechanisms of IS have not been systematically conducted.

This study aimed to review all published animal and clinical metabolomics studies on IS published from 2005 to 2024. By summarizing the research progress on the use of metabolomics in IS, this study aimed to systematically identify promising biomarkers of IS and the potential relationships between biomarkers and mechanisms such as excitotoxicity, oxidative stress, inflammation, and energy metabolism, which will potentially facilitate the discovery of biomarkers for predicting and diagnosing IS as well as distinguishing its etiology, assessing its prognosis, and elucidating its pathogenesis.

## Methods

### Literature review

We screened articles related to IS and metabolites included in PubMed between 2005 and 2024 April by using terms such as “metabolomics,” “metabonomics,” “biomarker,” “metabolome,” “metabolic profiling,” “metabolic signature,” “metabolic biomarker,” or “metabolic profile” alongside “stroke,” “cerebrovascular attack,” “CVA,” “cerebral ischemia,” “cerebral apoplexy,” “acute cerebrovascular accident,” or “acute cerebrovascular disease.” To obtain a more comprehensive metabolite database, some terms, such as “rodent,” “rat,” or “mouse,” were additionally included in our prophase work.

### Inclusion and exclusion criteria

Two authors (JS and CX) independently screened and selected relevant studies in accordance with the inclusion criteria. Disagreements at each step were resolved through discussion with all authors. Articles that met the following criteria were included:

1. Articles with full text

2. Articles with the title or abstract related to metabolism

3. Articles with keywords that included stroke or IS

4. Articles primarily discussing intracerebral hemorrhage

Review articles and articles without clear biomarker selection criteria were excluded

### Data extraction

The following data elements pertaining to the selected articles were extracted:

1. Title, first author, and publication year

2. Type and grouping method of study design, specimens, analytical platform

3. The metabolite showing significant changing trends and the trends themselves

**Figure 1:**
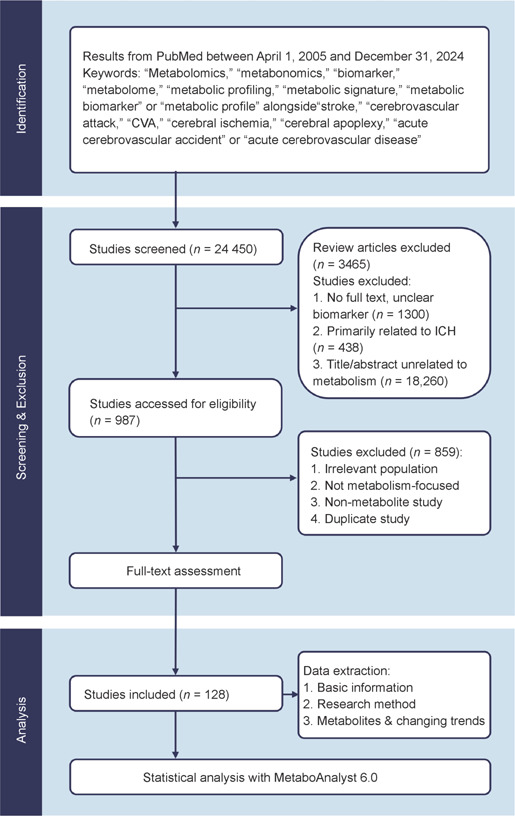
Flowchart of the study. ICH: Intracerebral hemorrhage.

The specific screening process is shown in **Figure 1**.

### Classification of metabolites

We conducted differential screening of metabolites across various samples and subsequently classified them into “positive” and “negative” categories based on their implications for stroke prognosis and the area under the curve values (Zhang et al., 2024). If the elevation of a specific metabolite was clearly linked to a favorable stroke prognosis, it was designated as a positive metabolite; if it was negatively correlated, it was labeled as a negative metabolite. A metabolite’s trend was deemed consistent when it exhibited the same directional change over two-thirds of the reviewed studies.

### Statistical analysis

The findings of metabolic profiling showing significant changing trends in the selected articles were extracted and illustrated. Since various articles used different compound IDs, we merged metabolites with identical chemical structures and retained only one ID for each metabolite. The metabolic pathway analysis and enrichment analysis were performed using MetaboAnalyst 6.0 (https://www.metaboanalyst.ca). Due to the lack of statistical information for metabolites needed to perform a meta-analysis, we utilized the Amanida package in R (http://github.com/mariallr/amanida) for a comprehensive meta-analysis of metabolomics data (Llambrich et al., 2021). Data inputs included metabolite names, *P*-values, fold-changes, and study sizes. Amanida’s weighted *P*-value approach, adapted from Fisher’s method, and log-transformed fold-changes were employed for assessing statistical significance and homogeneity, respectively. For the meta-analysis, the standardized mean difference (SMD) for potential biomarkers in the assessment of IS was calculated using Review Manager software version 5.4 (The Cochrane Collaboration, Copenhagen, Denmark), comparing patients with and without IS. A fixed- or random-effects model was used after analyzing the Q statistic, while the I^2^ method was used to evaluate heterogeneity. The statistical significance level was set at *P* < 0.05.

## Results

### Metabolites identified in middle cerebral artery occlusion mouse models

A total of 43 studies using middle cerebral artery occlusion (MCAO) rodent models were identified, which included 16 studies employing the MCAO mouse model (Sumbria et al., 2011; Alf et al., 2012; Berthet et al., 2014; Sheth et al., 2015; Abe et al., 2018; Cambray et al., 2018; Golovko and Golovko, 2018; Chen et al., 2019, 2023; Lepore et al., 2020; Tanaka et al., 2020; Krupska et al., 2021; Li et al., 2021a; Zheng et al., 2021; Mottahedin et al., 2023; Hasseldam et al., 2024) and 28 studies based on the MCAO rat model (van der Zijden et al., 2008; Paik et al., 2009; Lee et al., 2012; Yang et al., 2012; Gao et al., 2013; Kimberly et al., 2013; Wang et al., 2014; Sheth et al., 2015; Yan et al., 2015a, b; Liu et al., 2016b, 2018, 2020; Shin et al., 2016; Ivanov et al., 2017; Ruan et al., 2017; Baranovicova et al., 2018; Huang et al., 2018, 2023; Li et al., 2018; Geng et al., 2019; Wesley et al., 2019; Cheng et al., 2020; Rashad et al., 2020; Wu et al., 2021; Kong et al., 2022a; Zhang et al., 2022b; Dieu et al., 2023). Among the 16 studies utilizing the MCAO mouse model, 12 utilized mouse brain tissue samples, three employed plasma samples, and the remaining study utilized cerebrospinal fluid samples. The studies employing brain tissue samples included one study that compared metabolic alterations between mouse and human brain tissue (**Additional Table 1**). Notably, nine studies used mass spectrometry (MS), of which seven employed liquid chromatography mass spectrometry (LC-MS), one utilized capillary electrophoresis time-of-flight mass spectrometry, and one utilized both LC-MS and gas chromatography time-of-flight mass spectrometry. Additionally, one study employed microdialysis, one used matrix-assisted laser desorption/ionization-mass spectrometry imaging, three used magnetic resonance spectroscopy, one employed magnetic resonance imaging, and one study used high-performance liquid chromatography-fluorescence detection (**Additional Figure 1D**).

**Additional Table 1:** Summary of metabolomics studies in mice

Title	Published year	Study design	Specimens	Analytical platform	Up-regulated metabolites	Down-regulated metabolites
Acute depression of energy metabolism after microdialysis probe implantation is distinct from ischemia-induced changes in mouse brain	2011	Case-control	MCAO mice, cerebrospinal fluid	Microdialysis	Lactate, Glutamate, Glycerol	Glucose
High-resolution spatial mapping of changes in the neurochemical profile after focal ischemia in mice	2012	Case-control	MCAO mice, brain	1H MRS	Lactate, γ-Aminobutyric acid	N-acetyl aspartate, Taurine, Glutamate
N-acetyl aspartate levels early after ischemic stroke accurately reflect long-term brain damage	2024	Case-control	MCAO mice, brain	1H-MRS		N-acetyl aspartate
Metabolic fingerprints discriminating severity of acute ischemia using in vivo high-field 1H magnetic resonance spectroscopy	2020	Case-control	MCAO mice, brain	1H-MRS	Creatine, γ-Aminobutyric acid, Glycine, Lactate, Acetate	Glucose, *Myo*-inositol, N-acetyl aspartate, Phosphocreatine, Taurine, Alanine, Ascorbate, Aspartate, Glutamine, Glutathione, Macromolecule, Phosphorylcholine, Phosphorylethanolamine
Non-invasive diagnostic biomarkers for estimating the onset time of permanent cerebral ischemia	2014	Case-control	MCAO mice, brain	MRI	Creatine, γ-Aminobutyric acid, Glycine, Lactate, Acetate, Phosphorylethanolamine	Glucose, *Myo*-inositol, N-acetyl aspartate, Phosphocreatine, Taurine, Alanine, Ascorbate, Aspartate, Glutamine, Glutathione, Phosphorylcholine
Hippocampal Sector-Specific Metabolic Profiles Reflect Endogenous Strategy for Ischemia-Reperfusion Insult Resistance	2021	Case-control	MCAO mice, brain	LC-QTOF-MS	Piperidine, Hydroxypyridine, Indole, Pipecolic acid, Leucine, Histidine, Phenylalanine, 3-Methylhistidine, Tryptophan, Adenosine, Guanosine monophosphate, Stearoylcarnitine, LysoPG 18:1 sn-1, LysoPE 22:6 sn-1, LysoPI 18:1 sn-1, PC 18:1/18:1, PE 20:4/22:6	Taurine, Citric acid, Carnosine, Acetylaspartylglutamic acid, Arachidonoylcarnitine, Eicoseneoylcarnitine, LysoPE 18:1 sn-1, LysoPE 20:4 sn-1, LysoPI 18:0 sn-1, PE 22:6/P-16:0, PE 16:1/22:6, PE 16:1/16:0, PE 22:6/18:1
Metabolomic analysis and mass spectrometry imaging after neonatal stroke and cell therapies in mouse brains	2020	Case-control	MCAO mice, brain	CE-TOF MS	Phenylalanine, XC0132, Cystathionine, Lysine, Ornithine, Valine, Leucine, 2’-Deoxycytidine, Putrescine, Isoleucine	Isethioinic acid, Asparagine, N-Acetylaspartic acid, XA0065, N-Acetylglutamic, acid, N-Acetylneuraminic acid, Succinic acid, Creatine, Glutamate, GDP-glucose
An imbalanced ratio between PC(16:0/16:0) and LPC(16:0) revealed by lipidomics supports the role of the Lands cycle in ischemic brain injury	2021	Case-control	MCAO mice, brain	UPLC-MS/MS	Lysophosphatidylcholine (16:0)	Phosphatidylcholine (16:0/16:0)
UPLC-QTOF/MS-Based Metabolomics Reveals the Protective Mechanism of Hydrogen on Mice with Ischemic Stroke	2019	Case-control	MCAO mice, brain	UPLC-QTOF/MS	LysoPE(0:0/18:1), DL-2-Aminooctanoic acid, Citric acid, PalmitoyI-L-carnitine	oxidized Glutathione LysoPE(0:0/22:2) LysoPE(22:1) LysoPE(0:0/20:4) LysoPE(0:0/22:4) LysoPE(0:0/18:2) 2-Indolecarboxylie acid Acetylcarnitine PE(P-16:0) Glutathione LysoPC(16:1) Pyrrolidine L-Aspartic Acid N-acetylaspartate, Urolithin A-3-O-glucuronide Fumarprotocetraric acid LysoPS(18:1) LysoPE(0:0/22:5) Taurine LysoPC(0:0/20:4) N-Acetylaspartylglutamic acid LysoPC(16:0) PE(P-18:0) LysoPE(18:0) LysoPC(18:0)
Targeting succinate metabolism to decrease brain injury upon mechanical thrombectomy treatment of ischemic stroke	2023	Case-control	MCAO mice, brain	LC-MS/MS	Succinate	
Metabolomic Estimation of the Diagnosis and Onset Time of Permanent and Transient Cerebral Ischemia	2018	Case-control	MCAO mice, plasma	UHPLC-Q-T OF_ MS		Lactic Acid, 1,4-dideoxi-1,4-imino-D-arabinitol
Plasma Unesterified Fatty-Acid Profile Is Dramatically and Acutely Changed under Ischemic Stroke in the Mouse Model	2018	Case-control	MCAO mice, plasma	UPLC-MS	FFA 16:1n-7, FFA 16:1n-9, FFA 18:1n9, FFA 20:4n6, FFA 22:4n6, FFA 22:5n6, FFA 22:6n3	FFA 16:0, FFA 18:0
Metabolomic Analysis of Mouse Brain after a Transient Middle Cerebral Artery Occlusion by Mass Spectrometry Imaging	2018	Case-control	MCAO mice, brain	MALDI-MSI	Cer d18:1/18:0, Phosphocreatine	Creatine
The variation in the levels of 18 amino acids in the cortex and plasma of cerebral ischemia C57BL/6 mice	2021	Case-control	MCAO mice, brain	HPLC-FLR	Aspartic acid, Glutamic acid, Glycine, 4-Aminobutyric acid, Phenylalanine, Citrulline, Isoleucine, Leucine	Glutathione, Phenylalanine
Metabolomics profiling to characterize cerebral ischemia-reperfusion injury in mice	2023	Case-control	MCAO mice, brain	LC-MS/MS, GC-TOF-MS	Glucose-6-phosphate 1, Fructose-6-phosphate, Cellobiose 2, O-phosphonothreonine 1, Salicin, N-methyl-dl-alanine, Fructose 1, Alpha-aminoadipic acid, 2-deoxy-d-galactose 2, 2-hydroxyvaleric acid, N-acetylhistidine, Pentanenitrile, Histamine, Trans-hexadec-2-enoyl carnitine, 4-(diaminomethylideneamino)butan oic acid, (2s)-pyrrolidine-2-carboxylic acid, 2,4-dioxo-1h-pyrimidine-6-carboxyl ic acid	Cohibin C, 1-(sn-glycero-3-phospho)-1d-myo-inositol, 2- [methyl-(n ’-phosphonocarbamimidoyl)amino] ac etic acid, Myo-inositol, 3-aminopropionitrile 1, Erythrose 2, Threoninyl-arginine, (4s,5r,6r)-5-acetamido-2,4-dihydroxy-6-[(1r,2r)-1, 2,3-trihyroxypropyl]oxane-2-carboxylic acid, D-ribulose 5-phosphate, 2-hydroxypyridine, Indoxyl sulfate
Targeted Lipid Profiling Discovers Plasma Biomarkers of Acute Brain Injury	2015	Case-control	MCAO mice, plasma	HPLC-MS	SM 34:0, SM38:3, SM38:1, SM 37:1, SM 32:1, SM 36:3, SM 42:2, Cer 40:0	

**Figure 2:**
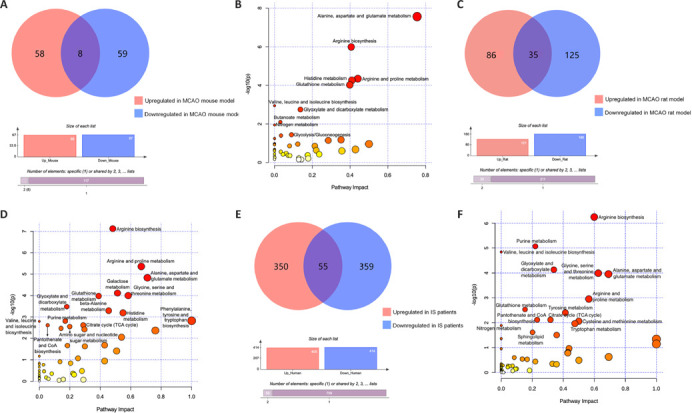
Venn diagrams and pathway analysis of potential biomarkers of IS. (A) Significantly changed metabolites in the published MCAO mouse model studies. (B) Pathway analysis of metabolites in the MCAO mouse model. (C) Significantly changed metabolites in the published MCAO rat model studies. (D) Pathway analysis of metabolites in the MCAO rat model. (E) Significantly changed metabolites in the published clinical studies. (F) Pathway analysis of metabolites in patients with ischemia. IS: Ischemic stroke; MCAO: middle cerebral artery occlusion.

In the MCAO mouse model studies, a total of 125 significantly altered (*P* < 0.05) metabolites were identified (**Figure 2A**). In comparison with the control groups, the IS groups showed increased and decreased levels of 58 and 59 metabolites, respectively. The remaining eight metabolites (citric acid, creatine, glutamate, aspartic acid, lysophosphatidylcholine(16:0) [lysoPC(16:0)], phenylalanine, phosphocreatine, and phosphorylethanolamine) showed upregulated trends in some studies and the opposite trends in others. Among these eight metabolites, aspartic acid, phosphocreatine, and phosphorylethanolamine specifically showed notable changes in the MCAO mouse model.

### Metabolites identified in middle cerebral artery occlusion rat models

Of the 28 studies employing the MCAO rat model, 14 used rat brain tissue, 11 used plasma samples (**Additional Table 2**), four employed serum samples, two employed cerebrospinal fluid samples, and one study each used liver tissue, heart tissue, and fecal samples. Furthermore, six studies set up diverse sample types for comparison, two compared brain and plasma metabolic alterations, and one each compared brain tissue, plasma, and liver tissue samples; plasma and cerebrospinal fluid samples; human urine and plasma samples; and heart tissue and plasma samples. The studies employed 12 types of platforms for metabolic alteration analysis, including MS (12 studies, including nine with LC-MS and three with GC-MS), high-performance liquid chromatography (two studies), magnetic resonance imaging (two studies), magnetic resonance spectroscopy (two studies), nuclear magnetic resonance (NMR) (six studies), enzyme-linked immunosorbent assay (one study), and matrix-assisted laser desorption/ionization-mass spectrometry imaging (one study) (**Additional Figure 1D**).

In the MCAO rat model studies, a total of 246 significantly altered (*P* < 0.05) metabolites were identified (**Figure 2C**), including 86 and 125 metabolites showing elevated and reduced levels in the IS group, respectively. The remaining 35 metabolites exhibited differing changing trends across studies; these metabolites were primarily related to valine, leucine, and isoleucine biosynthesis, glyoxylate and dicarboxylate metabolism, glycine, serine, and threonine metabolism, alanine, aspartate, and glutamate metabolism, cysteine and methionine metabolism, and the citrate cycle (tricarboxylic acid [TCA] cycle) (**Figure 2D**).

### Metabolites identified in patients with ischemic stroke

A compilation of 93 articles on patients with IS formed the database used for this review (**Additional Table 3**). Among these 93 studies, 79 employed a case-control design, three were prospective cohort studies, two each were retrospective and prospective studies, and the remaining studies included one prospective observational study, one observational prospective and multicenter study, one prospective and cross-sectional study, one longitudinal cohort study, and one multicenter prospective case-control study. The primary focus of 82 studies was metabolomics analysis discovery, while 11 studies were related to clinical outcomes. Notably, 39 of the 93 studies employed IS patient plasma samples (including one study utilizing both plasma and urine samples and one that employed human plasma and brain tissue samples), 31 utilized serum samples (including one study that utilized human serum and cerebrospinal fluid samples), 13 employed blood samples, nine used urine samples (including one study that compared IS patient urine samples with MCAO rat plasma samples), and one used dried blood spot specimens (**Additional Figure 1C**). MS-based analysis platforms were selected in 54 studies.

From the 93 studies included in our database, we identified 764 significantly altered (*P* < 0.05) metabolites in patients with IS, which encompassed 405 metabolites that were specifically reported to be elevated in patients with IS, 414 metabolites that were exclusively reported to be diminished in patients with IS, and 55 metabolites that showed varying trends (**Figure 2E**). The 55 metabolites exhibiting inconsistent trends across various studies were predominantly associated with the biosynthesis of phenylalanine, tyrosine, and tryptophan as well as with linoleic acid metabolism and the metabolism of alanine, aspartate, and glutamate (**Figure 2F** and **Additional Tables 4** and **5**).

**Additional Table 4:** Integration of metabolite names

20-COOH-Leukotriene B4	20-COOH-Leukotriene B4 20-Carboxyleukotriene B4
Tryptophan	L-Tryptophan Tryptophan
Methionine	L-Methionine Methionine
Leucine	L-Leucine Leucine
Isoleucine	L-Isoleucine Isoleucine
Cysteine	L-Cysteine Cysteine
Homocysteine sulfinic acid	L-Homocysteine sulfinic acid Homocysteine sulfinic acid
Proline	L-Proline Proline
Arginine	L-Arginine Arginine
Phenylalanine	L-Phenylalanine Phenylalanine
Valine	L-Valine Valine
Hompcysteine	L-Hompcysteine Hompcysteine
Cysteine sulfinic acid	L-Cysteine sulfinic acid Cysteine sulfinic acid
Lysine	L-Lysine Lysine
LysoPC(18:0)	LysoPC(18:0) LysoPC(0:0/18:0)
LysoPE (22:0)	LysoPE (0:0/22:0) LysoPE (0:0/22:0)
Homoarginine	L-Homoarginine Homoarginine
Tyrosine	L-Tyrosine Tyrosine
Thyroxine	L-Thyroxine Thyroxine
Acetylcarnitine	L-Acetylcarnitine Acetylcarnitine
Carnitine	L-Carnitine Carnitine
Xylulose	L-Xylulose Xylulose
Palmitoylcarnitine	L-Palmitoylcarnitine Palmitoylcarnitine
Glutamine	L-Glutamine Glutamine
Citrulline	L-Citrulline Citrulline
Aspartate	L-Aspartate Aspartate
Abrine	L-Abrine Abrine
Pipecolic acid	L-Pipecolic acid Pipecolic acid
Serine	L-Serine Serine
Threonine	L-Threonine Threonine
Aspartic acid	L-Aspartic acid Aspartic acid
Glutamic acid	L-Glutamic acid Glutamic acid
Pipecolinic Acid	D-Pipecolinic Acid Pipecolinic Acid
Ribose	D-Ribose Ribose

**Additional Table 5:** Metabolites with varying trends in patients with ischemic stroke and related pathway

55 metabolites with varying trends in ischemic stroke patients	Pathway
Valine	Valine, leucine and isoleucine biosynthesis;Pantothenate and CoA biosynthesis
Threonine	Valine, leucine and isoleucine biosynthesis; Glycine, serine and threonine
Isoleucine	Valine, b leucine h and isoleucine biosynthesis
Leucine	Valine, leucine and isoleucine biosynthesis
Dopamine	Tyrosine metabolism
2-Aminophenol	Tryptophan metabolism
Presqualene diphosphate	Steroid biosynthesis
Hypoxanthine	Purine metabolism
Inosine	
ITP	
Taurine	Primary bile acid biosynthesis; Taurine and hypotaurine metabolism
Cholesterol	Primary bile acid biosynthesis; Steroid biosynthesis
Glycocholate	Primary bile acid biosynthesis
Phenylalanine	Phenylalanine, tyrosine and tryptophan biosynthesis; Phenylalanine metabolism; Tyrosine metabolism
Tyrosine	Phenylalanine, tyrosine and tryptophan biosynthesis;Phenylalanine metabolism
Phenylacetylglutamine	Phenylalanine metabolism
Xylose	Pentose and glucuronate interconversions
Dephospho-CoA	Pantothenate and CoA biosynthesis
5-Sulfosalicylate	Naphthalene degradation
Lysine	Lysine degradation; Biotin metabolism
Carnitine	Lysine degradation
9-KODE	Linoleic acid metabolism
Linoleic acid	
Tryptophan	Glycine, serine and threonine metabolism; Tryptophan metabolism
Serine	Glycine, serine and threonine metabolism; Cysteine and methionine metabolism
Creatine	Glycine, serine and threonine metabolism; Arginine and proline metabolism
Choline	Glycine, serine and threonine metabolism
(9Z)-Hexadecenoic acid	Fatty acid biosynthesis
Stearic acid	
Methionine	Cysteine and methionine metabolism
Caffeine	Caffeine metabolism
3-Hydroxybutyric acid	Butanoate metabolism
Arachidonic acid	Biosynthesis of unsaturated fatty acids
Glutamine	Arginine biosynthesis; Alanine, aspartate and glutamate metabolism; Purine metabolism; Nitrogen metabolism
Aspartate	Arginine biosynthesis; Alanine, aspartate and glutamate metabolism; Pantothenate and CoA biosynthesis
2-Oxoglutarate	Arginine biosynthesis; Alanine, aspartate and glutamate metabolism; Butanoate metabolism; Citrate cycle (TCA cycle)
Citrulline	Arginine biosynthesis
Creatinine	Arginine and proline metabolism
Proline	
Succinate	Alanine, aspartate and glutamate metabolism; Butanoate metabolism; Citrate cycle (TCA cycle)
Alanine	Alanine, aspartate and glutamate metabolism
4-Methylumbelliferyl acetate	
9 10-DHOME	
Anandamide	
Androsta-1 4-diene-3 17-dione	
Anisole	
Benzyl alcohol	
Betanin	-
Catechol	
Cer(d18:1/22:0)	
Indole	
Pipecolic acid	
Salicyluric acid	
trans trans-Farnesyl diphosphate	
trans-o-Hydroxybenzylidenepyruvate	

### Middle cerebral artery occlusion mouse model *versus* middle cerebral artery occlusion rat model

**Figure 3:**
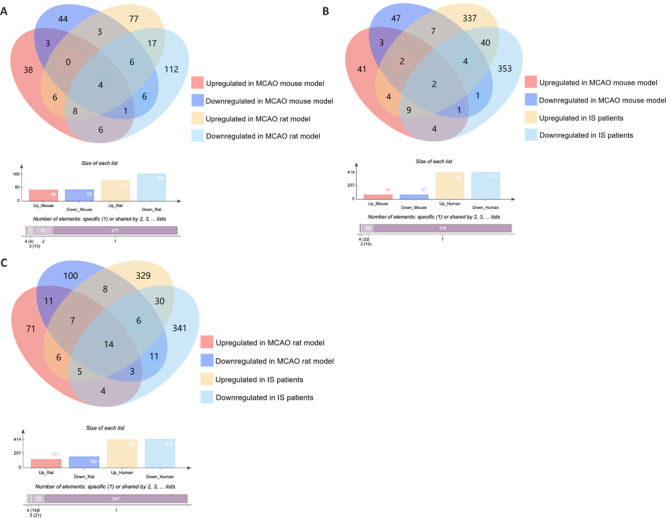
Venn diagrams of potential biomarkers of IS between pairs of groups. (A) MCAO mouse model *versus* MCAO rat model. (B) Ischemic patients *versus* MCAO mouse model. (C) Ischemic patients *versus* MCAO rat model. IS: Ischemic stroke; MCAO: middle cerebral artery occlusion.

Comparisons of the findings obtained in mouse and rat model experiments identified six metabolites as upregulated in both models (glutamic acid, glycerol, glycine, lysine, pipecolic acid, putrescine), and six as downregulated in both models (ascorbate, aspartate, LysoPC(18:0), N-acetylaspartate, N-acetylneuraminic acid, phosphorylcholine) (**Figure 3A**). The good reproducibility of these 12 metabolites may suggest that they play vital roles in the IS process.

In addition, four metabolites, namely, glutamate, creatine, citric acid, and phenylalanine, were discerned in four groups due to their divergent changing trends.

### Patients with ischemic stroke *versus* middle cerebral artery occlusion mouse model

Comparisons of the findings obtained for patients with IS and the MCAO mouse models revealed four metabolites (cer(d18:1/18:0), glutamic acid, glycerol, lactate) demonstrating a comparable upregulation, while lysoPC(18:0) was found to be downregulated (**Figure 3B**).

### Patients with ischemic stroke *versus* middle cerebral artery occlusion rat model

Comparisons of the findings obtained for patients with IS and the MCAO rat models showed six metabolites with comparable augmentation and seven metabolites exhibiting reductions (**Figure 3C**). The metabolites that showed reductions included 3-hydroxypropyl mercapturic acid, adenine, adenosine, adenosine monophosphate (AMP), adenosine triphosphate (ATP), LysoPC(16:0), LysoPC(18:0), malic acid, nicotinamide, phosphatidylcholine, and pyroglutamic acid. Conversely, the metabolites that showed increased levels were 5-aminoimidazole-4-carboxamide, asparagine, 3-hydroxybutyrate, glutamic acid, glycerol, lysophosphatidylethanolamine (24:0) (LysoPE(24:0)), and pseudouridine.

### Characterization of the screened metabolites into positive or negative

We further isolated the metabolites that demonstrated comparable results to patients with IS and classified them on the basis of their correlation with stroke prognosis and the area under the curve value. Ultimately, among 19 potential biomarkers, three were labeled as positive and two were tagged as negative (**Table 1**).

**Table 1:** Metabolites regarded as potential ischemic stroke biomarkers

Metabolite name	Regulated type	Area under the curve
3-Hydroxybutyrate	↑	–
5-Aminoimidazole-4-carboxamide	↑	–
Pseudouridine	↑	–
Cer (d18:1/18:0)	↑	–
Glutamic acid	↑	–
Glycerol	↑	–
LysoPC (18:0)	↓	–
3-Hydroxypropyl mercapturic acid	↓	–
Adenine	↓Positive	0.677
Adenosine monophosphate	↓	–
Adenosine triphosphate	↓	–
Malic acid	↓Positive	0.669
Nicotinamide	↓Positive	0.653
Phosphatidylcholine	↓	–
Pyroglutamic acid	↓Negative	0.753
Acetylcarnitine	↓	–
LysoPE (18:2)	↓Negative	0.785
LysoPE (20:4)	↓	–
LysoPC (20:4)	↓	0.784

### Comparisons among the three groups

**Figure 4:**
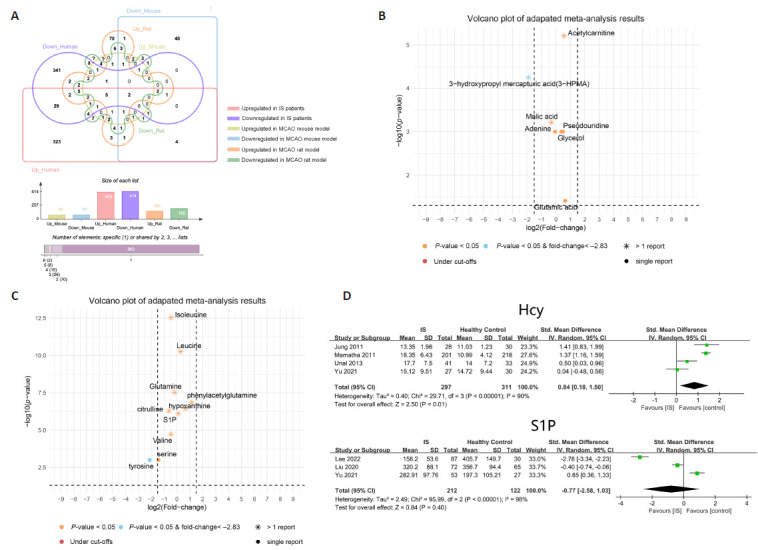
Venn diagrams and meta-analysis of potential biomarkers of IS in the three groups. (A) Venn diagram of comparison of the three groups. (B) Meta-analysis of 19 metabolites. (C) Meta-analysis of the most frequent metabolites. (D) Meta-analysis of homocysteine and S1P. Hcy: Homocysteine; IS: ischemic stroke; MCAO: middle cerebral artery occlusion.

Several metabolites exhibited similar outcomes to human sample metabolic analysis, indicating their high reproducibility, ease of detection, and considerable stability, which are essential for foundational research and represent promising stroke biomarker potential. Consequently, we compared the findings obtained in patients with IS with those obtained in MCAO rodent models, and ultimately identified two metabolites (glutamic acid and glycerol) that were upregulated in all models, and one (LysoPC(18:0)) that was downregulated across all models (**Figure 4A**).

The results of the Amanida meta-analysis of metabolomics in IS studies are shown in **Figure 4B** and **C**. Among the 19 metabolites, top dysregulated metabolites (vote score of ≥ 2.83 or ≤ –2.83) were selected as the consensus panel of meta-metabolites (**Figure 4A**). An Amanida analysis of the top 12 metabolites identified by frequency in the database was performed (**Figure 4C**). The panel contained four upregulated metabolites, including glutamic acid, glycerol, pseudouridine, and acetylcarnitine, as well as three downregulated metabolites, including 3-hydroxypropyl mercapturic acid, adenine, and malic acid. **Figure 4D** shows the results of meta-analysis of the potential biomarkers between the IS and healthy control groups according to the database. The homocysteine level significantly differed between the IS and the healthy control groups (SMD = 0.84, 95% confidence interval [CI] = 0.18–1.5, *P* = 0.01). However, the S1P level did not differ significantly between the groups (SMD = –0.77, 95% CI = –2.58–1.03, *P* = 0.40).

We further investigated pathway analysis of three models (**Additional Tables 1–3**), which implied that the IS metabolic pathway may be more represented by the pathways of alanine, aspartate, and glutamate metabolism and the TCA cycle. A comparison of the top 25 pathways indicated that the steroid hormone biosynthesis pathway identified in patients with IS was absent in the rodent models (**Additional Figure 2**). This divergence may be attributed to the predominant sex of the animals chosen for experimentation or the detection threshold limitations imposed by the instrumentation, highlighting the need for animal models that more closely mimic the pathophysiological conditions of cerebral stroke.

Moreover, a comparison of pathway analysis between the MCAO mouse and rat models revealed that the pathways enriched in the rat model more closely aligned with those in patients with IS (**Figure 2B**, **D**, and **F**). Thus, the reproducibility of stroke models in the rat model was superior to that in the mouse model.

### Relationship between biomarkers and mechanisms of ischemic stroke

#### Excitotoxicity

As the first reported and deeply studied molecular mechanism underlying IS, the “excitotoxic” concept primarily focuses on the role of depolarization underlying glutamate neurotoxicity and implies that the toxic action is mediated through N-methyl-D-aspartate receptors (Rothman and Olney, 1986). As a major excitatory neurotransmitter in the central nervous system, glutamic acid is the most abundant free amino acid in the central nervous system and plays a pivotal role in numerous metabolic pathways. After ischemic brain injury, glutamate acts as a critical transmitter in the degeneration of neurons involved in signal transmission (Lai et al., 2014). Specifically, under conditions of energy failure, glutamate is quickly released or taken up again and then acts on N-methyl-D-aspartate receptors, which could contribute to the overexpression of the downstream signaling pathways and cause a massive extracellular influx of calcium and its subsequent release from intracellular stores (Daniele et al., 2021). Emerging evidence suggests that sustained N-methyl-D-aspartate receptor overactivation and Ca^2+^ overload can result in elevated levels of free radicals and nitric oxide and the activation of lipases and proteases, and is closely related to mitochondrial dysfunction, consequently causing imbalance of energy metabolism (Qin et al., 2022; Shen et al., 2022). According to our metabolic pathway analysis, glutamate and glutathione metabolism are closely related to the pathological mechanism underlying IS. Gao et al. (2013) found that the levels of glutamine, alanine, aspartate, phenylalanine, and homocysteine all increased at 12 and 24 hours after MCAO in comparison with those in sham rats. Previous studies have shown that the concentration of the aromatic AA phenylalanine and its metabolite tyrosine were higher among patients with IS (Liu et al., 2015). Kagiyama et al. (2004) indicated that l-phenylalanine selectively and reversibly depressed the functioning of the glutamate receptor at excitatory synapses from rats or mice, causing a compensatory response to neurotoxic quantities of glutamate. Additionally, glutamate levels have been found to be higher in patients with IS and those with a high risk of progressing to IS than the control groups (Zheng et al., 2016; Wang et al., 2017). Pyroglutamic acid, also referred to as 5-oxoproline, serves as an intermediate in the metabolic pathway of glutathione. In the metabolic pathway of glutathione, gamma-glutamyl transpeptidase catalyzes the breakdown of glutathione into gamma-glutamyl amino acids. This is followed by the action of gamma-glutamyl cyclotransferase, which cyclizes the intermediate to produce pyroglutamic acid. Finally, under the catalysis of 5-oxoprolinase, pyroglutamic acid is converted into glutamic acid (Gamarra et al., 2019). Nevertheless, basic research elucidating the specific molecular mechanisms underlying this phenomenon is still lacking.

#### Oxidative stress

Oxidative stress is defined as the imbalance between high rates of oxidation and inadequate ability to clear reactive oxygen species (ROS) such as superoxide anions, hydroxyl radicals, and hydrogen peroxide (Sun et al., 2018; Jurcau and Ardelean, 2022). Although restoration of blood supply after IS is indispensable to aerobic metabolism, excess supply of oxygen could also lead to the production of ROS, which can cause protein denaturation, lipid peroxidation, DNA mutations, and even activate specific necrosis signaling cascades (Lévy et al., 2019; Su et al., 2019; Jurcau and Ardelean, 2022). Under the circumstance of the focal hypoxic region, the anaerobic pathway is the primary approach to produce energy instead of glucose metabolism, which contributes to the overgeneration of lactate and uric acid (UA) (Zhu et al., 2022).

Lactate is an important marker for ischemia and the subsequent hypoxic or anoxic conditions (Baranovicova et al., 2023). Wang et al. (2017) found that, in comparison with the control group, patients showed elevated lactate levels. The existing literature has also identified distinguishable increments in lactate levels in the brain tissue, cerebrospinal fluid, and serum of patients with acute IS (Wang et al., 2017). Furthermore, Chen et al. (2022) conducted a control study with a larger sample size, enrolling 400 patients with minor IS and 210 healthy controls, and obtained metabolomic results using magnetic resonance and untargeted metabolomics techniques. Their results showed that the serum lactate level in the minor IS group were higher than those in the control group. As a product of glycolysis, lactate is synthesized from pyruvate through lactate dehydrogenase and plays a crucial role in both physiological and pathological processes. However, it is also increasingly recognized as a signaling molecule that enhances cell survival. Recent studies have explored the neuroprotective effects of lactate against oxidative stress, revealing that lactate activates antioxidant mechanisms and pro-survival pathways through the production of nicotinamide adenine dinucleotide and a reduction in intracellular pH (Cerina et al., 2024). Additionally, Tauffenberger et al. (2019) demonstrated that l-lactate boosts cellular defense mechanisms, including the unfolded protein response and the activation of nuclear factor erythroid 2-related factor 2, by inducing a mild increase in ROS levels. This ROS surge activates antioxidant defenses and pro-survival pathways such as phosphoinositide 3-kinase (PI3K)/protein kinase B (AKT) and endoplasmic reticulum (ER) chaperones. As for IS, lactate has been found to be associated with neuronal cell death and the prognosis of patients after a cerebral ischemic event (Muñoz Maniega et al., 2008; Cvoro et al., 2009).

Under hypoxic conditions, DNA and AMP can produce hypoxanthine, which can be subsequently oxidized to UA. As an antioxidant, UA plays a causative role in scavenging free radicals (Becker, 1993). A growing body of studies has proved that serum UA (SUA) levels are positively related to the risk of developing IS (Khalil et al., 2020). Khalil et al. (2020) conducted a case-control study enrolling 338 participants to show that individuals were more likely to develop IS with elevation of SUA levels in comparison with patients without stroke history. Interestingly, their sex-specific analysis indicated that this positive association was observed only in females. However, the sex-related differences in the influence of SUA in IS have been a topic of debate among published studies, and the intrinsic mechanisms have remained uncertain to date.

#### Inflammation

In the absence of oxygen and glucose, dying or dead cells in areas such as the ischemic core release various danger-associated molecular patterns that recruit various leukocytes and chemokines to the brain and exacerbate the inflammatory reaction (Sakai and Shichita, 2023). The immune cells that first respond to these changes are the microglia and neutrophils located in the brain (Kolaczkowska and Kubes, 2013). Subsequently, more neutrophils, lymphocytes, and monocytes are attracted by microglia (Liu et al., 2016a). Eventually, resolution of inflammation is achieved by elimination of inflammatory mediators.

**Figure 5:**
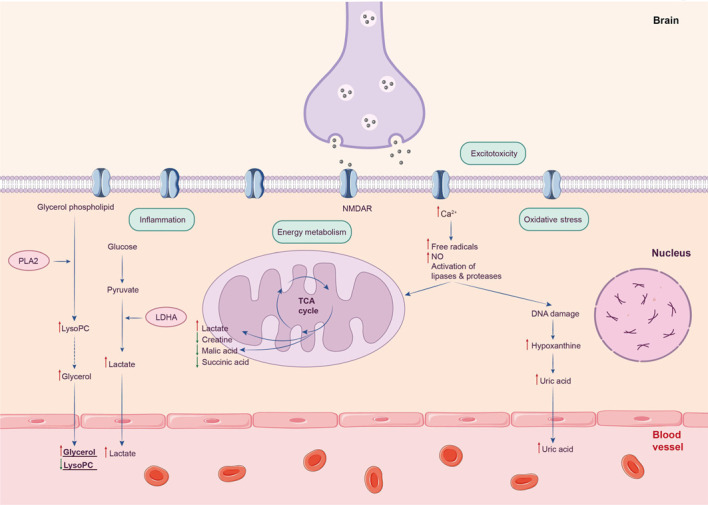
The relationship between metabolites and mechanisms of ischemic stroke. LDHA: Lactate dehydrogenase A; NMDAR: N-methyl-D-aspartate receptor; NO: nitric oxide; PLA2: phospholipase A2; TCA: tricarboxylic acid.

Studies have revealed that the inflammation after IS can substantially influence phospholipid metabolism and trigger noticeable changes in the lipid composition (Nakamura et al., 2020). Brain cells, which are characterized by a high density of lipids and are rich in a series of lipid classes, are more susceptible to stroke-induced inflammation. Some lipid mediators can alter the inflammatory process; thus, to some extent, their levels are correlated with stroke outcomes (Nakamura et al., 2020; Peng et al., 2024). On the other hand, previous studies have also shown that peripheral lipid metabolism can be influenced by ischemia events in animal experiments, which is potentially associated with a catabolic state induced by increasing release of inflammatory factors (Haley et al., 2017, 2020). Haley et al. (2017) observed long-term variations in metabolite levels, especially the lipid profile, at 60 days in MCAO mice. Their findings indicated a significant increase in the levels of plasma triglycerides and free fatty acids and the release of adipokines. A metabonomics study in patients with IS have reported similar lipid changes, including early increments in triglyceride levels (Ye et al., 2022). Lipids have shown promising potential, with lysophosphatidylcholine (LysoPC), phosphatidylcholine (PC), and lysophosphatidylethanolamine (LysoPE) being increasingly recognized as biomarkers of IS (Jové et al., 2015; Liu et al., 2017; Nakamura et al., 2020; Zheng et al., 2021; Ye et al., 2022; Lee et al., 2024). Moreover, spatial metabolomics research has revealed that in comparison with the sham surgery group in rats, the MCAO group shows significantly elevated levels of Cer(d18:1/16:0), LysoPC(16:0), LysoPC(18:1), and fatty acid (22:6) (FA-22:6) in the ischemic core region (Ban et al., 2024). Another study observed unique metabolic remodeling at different stages on the 7^th^ and 28^th^ days after stroke in mice. Over time, the concentrations of lysophosphatidylethanolamine (LPE) O-16:1 and LPE O-18:1 in the ischemic side of the somatosensory motor cortex region were found to be significantly higher than those on the contralateral side, suggesting that LPE may promote neuronal recovery in this area by stimulating neurite growth in cultured neurons (Wang et al., 2024a). Specifically, LysoPC represents a class of phosphorus-containing lipids that are currently reported to be associated with the occurrence and progression of coronary atherosclerosis (Chisolm and Chai, 2000). Characterized by the presence of both hydrophobic and hydrophilic groups, LysoPC is an amphiphilic, highly active agent capable of lysing cell membranes, leading to cell lysis (Liu et al., 1991). Unlike other phosphatidyl molecules, LysoPC maintains relatively high concentrations of approximately 140–150 μM in body fluids (Chen et al., 1997), and its levels can rise to the millimolar per liter level under conditions of hypercholesterolemia, facilitating the detection of its level variations in peripheral blood. The primary sources of endogenous LysoPC are the phospholipid bilayer of cell membranes and the PC on the surface of lipoproteins. Under the action of phospholipase A1 (PLA1) and phospholipase A2 (PLA2), PC glycerol is progressively hydrolyzed into LysoPC (**Figure 5**). Its metabolites can be converted into 3-phosphoglycerate through the action of lysophospholipases and other enzymes, thereby entering the lipid metabolism pathway to produce downstream lipids such as glycerol, triglycerides, and phosphatidic acid. PLA2 is a key enzyme affecting the generation of LysoPC; this enzyme requires millimolar levels of calcium ions for activation, which can significantly increase during the reperfusion process following IS, leading to superoxide action on membrane phospholipids (Li et al., 2021b; Zhang et al., 2022a). Its activity is also regulated by inflammatory factors, including interleukin (IL)-1, IL-6, and tumor necrosis factor-α, indicating that the increased secretion of inflammatory factors in IS can potentially enhance PLA2 activity. Nakamura et al. (2023) found that PLA2G2E in neurons surrounding the infarct area can generate dihomo-γ-linolenic acid. 15-Hydroxy-eicosatrienoic acid, a subtype of dihomo-γ-linolenic acid metabolites, plays a pivotal role in triggering autonomic neural repair in the brain, leading to improved functional outcomes in patients recovering from IS. LysoPC has also been reported to be intricately linked to inflammatory conditions, and it plays a crucial role in numerous signaling pathways that are fundamental to the progression of inflammatory responses (Hung et al., 2012; Shanta et al., 2012). Furthermore, preclinical studies encompassing both *in vivo* and *in vitro* methods have consistently illustrated that LysoPC possesses a promising protective role in ameliorating ischemic damage and curtailing neuronal demise in individuals with stroke (Blondeau et al., 2002). Liu et al. (2018) found that the concentrations of LysoPC(18:0) and LysoPC(16:0) in MCAO rats declined compared with the control groups in the serum of rats. Consistent with these findings, a clinical study by Huang et al. (2023) showed that LC-MS evaluation of the plasma concentrations of LysoPC could offer valuable insights into the management and rehabilitation phases of stroke care. A clinical study showed that the levels of LysoPC and PC were significantly related to the recovery of IS patients. LysoPC probably promoted long-term functional recovery by exerting an anti-inflammatory effect (Huang et al., 2022). The conversion of LysoPC back to PC is facilitated by the enzyme LysoPC acyltransferase, which operates with acyl-CoA as a substrate. The dynamic shifts in the concentration ratio between PC and LysoPC epitomize the body’s cyclical anabolic and catabolic processes for PC, which is an integral part of the Lands cycle (Wang and Tontonoz, 2019). An imbalance in the PC-LysoPC ratio has been shown to exacerbate the brain damage after ischemia in MCAO mice (Zheng et al., 2021). In addition, the crucial role of the proteins associated with LysoPE is widely acknowledged in various motility-associated processes, including angiogenesis and neurite extension (Moolenaar and Perrakis, 2011). LysoPE(18:2) and LysoPE(20:4) both showed decreased trends in the samples of patients with IS (Liu et al., 2017).

Reinke et al. (2013) conducted a study using ^1^H NMR and observed significant alterations in the concentration of glycerol. Consistent with these findings, another clinical research showed higher glycerol levels in the patient groups (Denihan et al., 2019). Similarly, using an ultra-performance liquid chromatography-tandem mass spectrometry (MS/MS) platform, a nested case-control study reported that cardinal shifts in lipid profiles during the initial phases of hypertensive stroke are predominantly linked to the metabolic pathways of glycerophospholipids and choline (Zeng et al., 2024). A weighted gene co-expression network analysis demonstrated a broad association between glycerophospholipids and glycerolipids with the occurrence of stroke. Glycerophospholipids, integral components of the neuronal cell membrane, play a crucial role in cellular recognition and the transduction of signals (Zeng et al., 2024). Clinical studies have revealed that the administration of glycerol, either intravenously or orally, can rapidly reduce ischemic cerebral edema in patients (Berger et al., 2005). Glycerol may also act as a protective agent against neuroinflammation by potentially providing energy to neurons in place of glucose or modulating the interactions between leukocytes and endothelial cells, ultimately improving cerebral blood flow and enhancing prognosis (Sloviter et al., 1966; Ishikawa et al., 1999). However, the existing metabolomic research on the role of glycerol in the context of stroke is limited. Additional studies are required to delve deeper into the relationship between glycerol and stroke, and to explore the clinical implications of glycerol as a potential biomarker.

#### Energy metabolism

Although the brain accounts for only 2% of body mass, it is responsible for approximately 20% of the total energy consumption (Mink et al., 1981). The lack of oxygen supply that follows arterial occlusion can result in disturbed energy metabolism. Many recent studies have suggested that changes in the levels of a variety of metabolites, including glucose, lactate, creatine/phosphocreatine, and the metabolites of the TCA cycle, are the most noticeable during disturbances of energy metabolism (Diaz and Raval, 2021; Jia et al., 2021; Sifat et al., 2022). The citrate cycle, also known as the Krebs cycle, is a paramount process for energy metabolism within mitochondria, accounting for the production of over 90% of the cellular energy supply (Pieczenik and Neustadt, 2007; Akram, 2014). After ischemic attacks, AMP levels in the cortex and hippocampus drop sharply, indicating a state of energy depletion in brain tissue. To sustain energy demands, mitochondria, as crucial bioenergetic organelles, drive metabolic reprogramming, shifting cells from aerobic to anaerobic metabolism. The accumulation of glucose and glycolytic intermediates is a prominent feature of the metabolic dysregulation induced by cerebral ischemia in rodents (Zhang et al., 2016). Luo et al. (2019) used 5-(diisopropylamino)amylamine derivatization-UHPLC-Q-TOF/MS to conduct a metabolomics study with cerebrospinal fluid samples from permanent MCAO model rats. They observed significant variance in the levels of citrate, malate, succinate, and isocitrate, of which citrate levels showed the most pronounced disparity between the MCAO and the high-dosage groups. Furthermore, Yu et al. (2021) utilized untargeted metabolomic analysis by LC-MS to evaluate plasma samples and observed that citric acid, trans-aconitic acid, l-Malic acid, and succinic acid were all remarkably downregulated in IS patients in contrast to healthy controls, indicating prominent alterations in the TCA cycle and further substantiating the significance of energy metabolism in IS. In a prospective cohort study, high-resolution non-targeted metabolomics technology was used to analyze serum samples from patients at risk of stroke and non-risk control participants. The findings revealed a decreasing trend in ATP and AMP levels within guanosine (Khan et al., 2020). Emerging findings suggest that mitochondria can serve as an effective target for reinstating cerebral blood flow following stroke. ATP, AMP, and adenosine diphosphate are capable of influencing cerebrovascular tone through their actions on plasmalemmal purinergic receptors (McIntosh and Lasley, 2012).

## Discussion

This review presents a novel systematic analysis of potential biomarkers and metabolic pathways implicated in IS, categorized by species. Our previous study showed that metabolites such as LysoPC(15:1) are related to neuroprotective mechanisms in the Glu-HT-22 model, highlighting the promising role of metabolomics in IS research (Ren et al., 2023). In this study, through a comparative examination of the similarities and differences among three species, the biomarkers and metabolic pathways with the greatest potential for clinical utility were discerned. Metabolites from various samples that demonstrated uniform trend patterns were then filtered. The screening process identified three positive and two negative metabolites. Moreover, our integrative analysis correlated the identified potential biomarkers with the mechanisms of IS, elucidating the relationships among them.

### Clinical significance of biomarkers

#### Biomarkers for predicting risk

Commonly acknowledged risk factors such as hyperlipidemia and diabetes mainly manifest as changes in the levels of metabolites. Consequently, monitoring metabolic changes directly is a more accurate approach than traditional approaches to predict the risk of IS development. Linoleic acid (LA) is recognized for its significant capacity to alleviate high blood pressure and stave off atherosclerosis (Hadipour et al., 2023). A large prospective observational study that followed up 68,659 participants proved that individuals with higher levels of LA in circulating blood and tissue had lower risk of incident cardiovascular disease, supporting dietary recommendations for preventing the occurrence of IS (Marklund et al., 2019). One recent study demonstrated the levels of LA in patients with stroke were lower than those in the control group, which is in line with prior research (Jiang et al., 2011; Kong et al., 2022b). Moreover, a recent study on lipid biomarkers selected potential diagnostic biomarkers, including PE(22:6/P-18:0), Cer 34:2, GlcCer(d18:0/18:0), DG 44:0, and LysoPC(16:0), and then established and validated a support vector machine model to predict the risk of IS (Ye et al., 2022).

In summary, the metabolomics approach stands out as a potent approach for forecasting the onset of stroke. This advancement could facilitate timely lifestyle modifications and risk factor reduction, thereby decreasing the incidence of stroke.

#### Biomarkers for diagnosis

The conventional classification of IS is principally based on the TOAST classification, which aims to facilitate individual treatment according to the specific etiology. In particular, previous studies have mentioned that the patients with large-artery atherosclerosis (LAA) subtype IS have worse outcomes and higher mortality, but the specific pathological mechanisms and target metabolites remain unclear (Sacco et al., 1991). Wang et al. (2020) inferred disturbed lipid and amino acid metabolism by performing non-targeted metabolomics analyses of the serum profiles of patients with the LAA and small-vessel occlusion subtypes. In addition, one recent targeted metabolomics study showed that six biomarkers, namely, lysine, serine, threonine, kynurenine, putrescine, and LysoPC(16:0), could distinguish the serum profiles in LAA and cardioembolic patients (Lee et al., 2024).

A multitude of observational and epidemiological investigations have substantiated the association of elevated homocysteine levels with the risk and severity of stroke and poorer patient outcomes (Fu et al., 2015; Wang et al., 2015; Zhou et al., 2021). Nevertheless, the designs of these studies typically did not account for the underlying etiologic subtypes of stroke. Consequently, the correlation between specific IS subtypes and homocysteine levels remains a subject of debate, with findings that are often inconsistent. Gungor et al. (2018) hypothesized that the serum homocysteine level may be associated with particular subtypes of atherothrombotic IS. By recruiting 262 stroke patients, they found a robust association between elevated homocysteine levels and the LAA subtype in young and middle-aged patients with IS. This correlation held significant even when accounting for a range of other potential risk factors. Thus, increased homocysteine levels in the bloodstream could be a contributing factor to the early development of symptomatic intracranial artery stenosis. Another recent study that included 805 participants demonstrated a positive relationship between plasma homocysteine concentrations and the LAA and small artery occlusion subtypes of IS, whereas no such correlation was observed with the cardioembolic subtype (Zhang and Zhang, 2023). Additional studies with a prospective, controlled approach are required to substantiate these links and evaluate the impact of homocysteine levels on the possibility of developing specific stroke subtypes.

#### Biomarkers for clinical outcomes

Studies of biomarkers for IS outcomes have mainly focused on hemorrhagic transformation (HT), infarct volume, poor stroke functional outcomes, mortality, early neurological deterioration, and infection. With the conventional application of anticoagulants and thrombolytics, the risk of HT has inevitably increased (Álvarez-Sabín et al., 2013). Gnofam et al. (2013) observed that increased baseline serum glucose levels caused increments in the occurrence rate of symptomatic HT (sHT) in non-diabetic patients treated with intravenous (iv) recombinant tissue plasminogen activator (rt-PA). Moreover, a prospective, multicenter, cohort study enrolling 2370 patients proved that admission hyperglycemia (within 3 hours after stroke onset) increased the rate of symptomatic intracranial hemorrhage at 90 days and enhanced major disability and death at 30 and 90 days, implying that the serum glucose level could serve as a promising biomarker to predict the possibility of symptomatic intracranial hemorrhage and deterioration of functional outcomes (Lin et al., 2018). With regard to the infarct volume, a previous study using diffusion-weighted imaging to calculate the lesion size showed that high plasma glutamate levels were closely linked to the infarct growth at 72 hours after acute hemispheric infarction. Moreover, the authors also noticed that the glutamate concentrations were higher in patients whose early neurological deterioration was poorer (Castellanos et al., 2008).

### Temporal and spatial variations in metabolites

Since some biomarkers may show fluctuating patterns in patients with IS, monitoring of temporal variations in biomarker levels can not only enhance the accuracy of outcome evaluations, but can also reflect the dynamic pathological process. Abe et al. (2018) reported that creatine levels decreased in hippocampal CA1 (CA1) from 1 hour to 24 hours after transient MCAO but increased in the caudoputamen. Moreover, in the cerebral cortex, creatine expression increased up to 1 hour after MCAO but reduced at 24 hours. Therefore, the timepoints as well as the location of brain tissue materials can influence the experimental results obtained for creatine expression.

Although the levels of glutamate showed decreasing and increasing trends in our rodent models, some studies have suggested that the glutamate concentration starts increasing at the onset of IS, peaks at a few hours after MCAO (Harpaz et al., 2017), and then begins to decrease (Harpaz et al., 2017). Furthermore, Yan et al. (2015a) summarized that the glutamate level peaks at 2 hours after MCAO, while Zhang et al. (2022b) showed an elevated level 3 hours after MCAO. Among clinical studies, the study mentioned above observed that the glutamate concentration continued to increase at 24 and 72 hours, but the quantitative value at 72 hours was close to that in patients with non-early neurological deterioration (Castellanos et al., 2008). Another recent study revealed that the serum levels of glutamate increased with a latency time of 121 minutes, which was consistent with the acute stage of glutamatergic excitotoxicity in the brain (Da Silva-Candal et al., 2021). The authors also found that glutamate levels of patients with latency time within 121 minutes were higher than other ranges, showing the existence of a time-dependent nature (Da Silva-Candal et al., 2021). Consequently, in the absence of solid evidence to examine precise timepoints, the exact correlation between the process of IS and glutamate concentration changes requires elucidation.

Hong et al. (2010) analyzed the concentrations of UA from blood samples of 88 patients with acute IS in the anterior circulation system after 48 hours, 7 days, and/or 14 days of admission and found that the levels of UA began to decline initially from the time of admission, reached the minimum at 48 hours, and then gradually increased until day 14, presenting an overall U-shaped pattern. This study identified a correlation between UA levels and the infarct volume as well as the vascular recanalization status, which reflected the eventual clinical outcome (Hong et al., 2010).

Nonetheless, the sequential alterations of biomarkers following the occurrence of IS still remain under-reported. Investigating the inconsistencies in metabolite variations between rodent models and clinical studies can reveal the complex interplay of contributing factors, including the timing of sample collection, sample types, analytical platforms, differences in the experimental design of MCAO models, and the inherent limitations of animal models.

### Limitations of metabolomics studies of ischemic stroke

Metabolomics studies can be mainly divided into non-targeted and targeted studies, both of which commonly use NMR and MS. Non-targeted metabolomics, which is employed in most of the studies in this field, identifies potential biomarkers by analyzing different metabolites from tested and healthy control groups. The targeted method validates the clinical significance of specific metabolites reported in previous research. In comparison with the non-targeted method, the targeted method is characterized by greater sensitivity and specificity, but it lacks the breadth of measurement achieved with the non-targeted approach (Au, 2018). Thus, the combination of non-targeted and targeted methods can enhance the accuracy of identification and the subsequent quantification of biomarkers.

As a comparatively promising and rapidly developing technology in omics-based methods, including genomics, transcriptomics and proteomics, metabolomics has the advantage of analyzing smaller numbers of metabolites. Additionally, metabolomics can allow analyses downstream of the central dogma and environmental changes and directly reflect the authentic phenotypes and medical condition (Shah et al., 2012). Recent reports have highlighted the potential of spatial metabolomics for detecting cellular metabolic states and their heterogeneity, identifying metabolic cell fate trajectories, and generating spatial maps of metabolic activity (Alexandrov, 2023). However, more experimental and computational innovations are required to target the current challenges, including enhancing sensitivity, achieving higher spatial resolution, and accelerating analysis. Moreover, the use of metabolomics has the same limitations as other omics-based technologies, including differences in sample-collection and recruitment strategies. In the field of genomics, single-cell sequencing is an emerging technique that has been shown to be capable of distinguishing the nucleotide and genome structure changes more specifically at a single-cell level (Yasen et al., 2020). Unfortunately, this technology shows a limited breadth of genomic coverage and amplification bias (Macaulay and Voet, 2014). Variability in gene expression is common even in cells with a homogeneous phenotype (Aevermann et al., 2018). Transcriptomics can detect the disease-related gene expressions and reveal the functions of certain cell more sensitively. Nevertheless, transcriptomics also shows sequencing bias, high error rates, and limited throughput. As the functional entities that perform activities, proteins play an indispensable role in the pathophysiological process of illness, and the phenotype is closer to the output of proteomics studies than those of genomics and transcriptomics. Consequently, proteomics is still the most widely used approach to explore novel biomarkers (Harpaz et al., 2017). However, the detection accuracy of two-dimensional gel electrophoresis and its resolution represent unresolved technological difficulties. Multiple studies have reported various statistical methods to analyze single-omics data, which would still ignore the interactions among molecular elements and potential pathways (Wang et al., 2024). The limitations of single-sequencing analysis represent a challenge to comprehensively capture the time-dependent molecular events following disease onset. Consequently, integromics, which refers to a comprehensive multi-omics approach, is anticipated to become a promising method to illuminate the pathological mechanism of IS (Montaner et al., 2020; Li et al., 2022).

Our study presents several innovative aspects. First, we have conducted a systematic screening and summarization of potential biomarkers and metabolic pathways from current research on three species: the mouse MCAO model, rat MCAO model, and patients with stroke. Despite extensive research using animal models, the clinical translation rate of IS drugs is disappointingly low, potentially due to the limited information regarding IS biomarkers. The biomarkers identified among these species not only show potential utility as clinical diagnostic markers, but also can be applied in basic experimental research to explore the mechanisms underlying IS. This comprehensive analysis is expected to facilitate the identification of biological targets that can improve the success rate of clinical translation. Second, we have elucidated the potential interplay between biomarkers and IS pathophysiology, which can guide the selection of appropriate models for basic research on specific biomarkers. Third, the clinical significance of biomarkers in IS has been summarized, highlighting their role in predicting risk, diagnosing specific subtypes, and assessing prognosis. Additionally, we have provided an innovative overview of temporal and spatial variations of IS biomarkers, offering insights for the selection of detection time points and sampling sites in future studies.

## Limitations

Although this systematic review was conducted meticulously, it has certain limitations. First, we mainly focused on the MCAO rodent models that were most widely used. Due to the differences in sample selection and analytical techniques across studies, we did not employ a unified set of specific clinical threshold values or receiver operating characteristic, positive predictive value, or negative predictive value thresholds to define the clinical significance of the biomarkers. Second, limitations occurred at both the study level (such as the risk of bias in individual studies) and the review level (such as the potential for incomplete retrieval of identified research and reporting bias). Future studies should further explore the cell-type specificity of these metabolites and their potential effects on glial and endothelial cells.

## Conclusion

In this study, we systematically reviewed 128 published articles on IS and metabolic profiling from 2005 to 2024. Subsequently, three positive (protective) biomarkers and two negative (detrimental) biomarkers were categorized on the basis of their influence on stroke outcomes and the area under the curve values among multiple groups, including mice, rats, and patients. Eventually, glutamic acid, glycerol, and LysoPC(18:0) emerged as potential biomarkers for IS prediction and intervention. Subsequently, the potential relationships of biomarkers and mechanisms of IS, including excitotoxicity, oxidative stress, inflammation, and energy metabolism, was elucidated. This study analyzed and identified promising biomarkers, which may yield critical targets and strategies for prediction, diagnosis, and prognostic evaluation of IS.

## Additional files:

***Additional Figure 1:*** *Categorization of metabolomics data: metabolites, specimen types and analytical platforms.*

Additional Figure 1 Categorization of metabolomics data: metabolites, specimen types and analytical platforms.
(A) Top frequency metabolites. (B) Specimen type of rodent model. (C) Specimen type of IS patients. (D) Rodent model analytical platform. (E) IS patients analytical platform. IS: Ischemic stroke.

***Additional Figure 2:*** *Pathway analysis of the three groups.*

Additional Figure 2 Pathway analysis of the three groups.
(A) Pathway analysis of metabolites on middle cerebral artery occlusion mouse model. (B) Pathway analysis of metabolites on middle cerebral artery occlusion rat model. (C) Pathway analysis of patients with ischemic stroke.

***Additional Figure 3:*** *A Venn diagram of the number of metabolomics literature in the three groups.*

Additional Figure 3 A Venn diagram of the number of metabolomics literature in the three groups.

***Additional Table 1:*** *Summary of metabolomics studies in mice.*

***Additional Table 2:*** *Summary of metabolomics studies in rats.*

Additional Table 2 Summary of metabolomics studies in rats

***Additional Table 3:*** *Summary of metabolomics studies in ischemic stroke patients.*

Additional Table 3 Summary of metabolomics studies in ischemic stroke patients

***Additional Table 4:*** *Integration of metabolite names.*

***Additional Table 5:*** *Metabolites with varying trends in patients with ischemic stroke and related pathway.*

## Data Availability

*All relevant data are within the paper and its Additional files.*
