# Supplementary material for: Advances in metabolomics of biomarkers for ischemic stroke: From bench to clinic
Source: Neural Regen Res. 2025 Jul 5;21(8):3588–97. doi: 10.4103/NRR.NRR-D-24-01384 (PMC13452661; doi:10.4103/NRR.NRR-D-24-01384)
Supplement: Supplementary file 1 [file NRR-21-3588_Suppl1.pdf]

Additional Table 2 Summary of metabolomics studies in rats

Title	Published year	Study design	Specimens	Analytical platform	Up-regulated metabolites	Down-regulated metabolites
Transcranial Direct-Current Stimulation Regulates MCT1-PPA-PTEN-LONP1 Signaling to Confer Neuroprotection After Rat Cerebral Ischemia-Reperfusion Injury	2022	Case-control	MCAO rats, cerebrospinal fluid	HPLC	propionic acid	
Local and systemic metabolic alterations in brain, plasma, and liver of rats in response to aging and ischemic stroke, as detected by nuclear magnetic resonance (NMR) spectroscopy	2019	Case-control	MCAO rats, brain	NMR	Lysine, Tryptophan, Glycine, Methionine, Isoleucine, Ethanolamine, Fumarate, Glycerol, Leucine, Pantothenate, Phenylalanine, Valine	Glutathione, Serine, NAA
			MCAO rats, liver		Choline, 3-Hydroxybutyric acid, Creatine, Taurine	Tyrosine, Glutathione, Tryptophan, Serine, Alanine, Histamine, PC
			MCAO rats, plasma		Lysine, Glycine, Isoleucine, ALCAR	Tyrosine, Methionine, Choline, Citrate, Proline, Threonine
Metabolite profiling identifies a branched chain amino acid signature in acute cardioembolic stroke	2013	Case-control	MCAO rats, Plasma and cerebrospinal fluid	LC-MS/MS	Xanthosine, Carnosine, Glutamate	Leucine, Isoleucine, Valine, Phenylalanine, Niacinamide
The free fatty acid metabolome in cerebral ischemia following human mesenchymal stem cell transplantation in rats	2009	Case-control	MCAO rats, plasma	CG-MS	Caproic acid (C6:0), Caprylic acid (C8:0), Decenoic acid(C10:1), Capric acid (C10:0), Lauric acid (C12:0), Myristoleic acid (C14:1), Myristic acid (C14:0), γ-Linolenic acid (C18:3n6), Eicosapentaenoic acid (C20:5n3), Docosahexaenoic acid (C22:6n3), Docsapentaenoic acid (C22:5n3), Erucic acid (C22:1), Nervonic acid (C24:1), Lignoceric acid (C24:0), Hexacosanoic acid (C26:0)	Palmitic acid (C16:0), Linoleic acid (C18:2n6), Arachidonic acid (C20:4n6), Eicosenoic acid (C20:1)

			MCAO rats, brain		Myristic acid (C14:0), Linoleic acid (C18:2n6), Stearic acid (C18:0), Eicosenoic acid (C20:1), Eicosanoic acid (C20:0), Docsapentaenoic acid (C22:5n3), Erucic acid (C22:1), Behenic acid (C22:0)	Palmitic acid (C16:0), Oleic acid (C18:1)
Restoration of Polyamine Metabolic Patterns in In Vivo and In Vitro Model of Ischemic Stroke following Human Mesenchymal Stem Cell Treatment	2016	Case-control	MCAO rats, brain	CG-MS	Putrescine, Cadaverine, *N*1-acetylspermidine, Spermidine	
Protective effects of Polygonum multiflorum on ischemic stroke rat model analysed by 1H NMR metabolic profiling	2018	Case-control	MCAO rats, brain	1H-NMR	Leucine, Isoleucine, Valine, 3-Hydroxyisobutyrate, 3-Hydroxybutyrate, Lactate, β-Alanine, Alanine, Lysine, Glutamine, Succinate, Methionine, Ethanolamine, Choline, sn-Glycero-3-phosphocholine, Taurine, Glucose, Glycine, Threonine, Uracil, Cytidine, Fumarate, Tyrosine, Anserine, Phenylalanine	GABA, Acetate, N-acetyl-Aspartate, Glutamate, Glutathion, Aspartate, Trimethylamine, Creatine, Malonate, Phosphocholine, Ascorbate, myo-Inositol, Inosine, UDP-galactose, UDP-glucose, Uridine, Guanosine, AMP, ADP, ATP, Carnosine, Nicotinamide, Oxypurinol, Hypoxanthine, NADH
Metabolomics study on the effects of Buchang Naoxintong capsules for treating cerebral ischemia in rats using UPLC-Q/TOF-MS	2016	Case-control	MCAO rats, plasma	UPLC-Q/TOF-MS	Cholic acid, Pseudouridine/Uridine, pyruvate, Taurine, Lactate, Glutamine, Alanine, Phenylalanine, Tryptophan, Histidine, Norepinephrine, 5-hydroxyindoleacetic acid, LysoPE(24:0), cytidine, creatine, Palmitoyl-l-carnitine, *N*-stearoylserine	Gly-Gln-Leu, Gly-Cys-Ala-Phe, PC(14:0/0:0), PE(17:0/0:0), PS(20:0/0:0), PC(14:1), LysoPE(0:0/16:0), LysoPE(20:1), LysoPE(0:0/24:1(15Z)), LysoPE(20:0/0:0), Acetone
Dynamic metabolites profile of cerebral ischemia/reperfusion revealed by (1)H NMR-based metabolomics contributes to potential biomarkers	2014	Case-control	MCAO rats, serum	1H-NMR	Ornithine, Glutamic acid, Glycine, Succinic acid, Malonic acid, Citric acid	L-Lactic acid, Pyruvic acid, Betaine, L-Serine, L-Alanine, L-Threonine, Acetic acid, L-Valine
Ultra Performance Liquid Chromatography/Quadrupole Time-of-Flight Mass Spectrometry-Based Metabonomics Reveal Protective Effect of Terminalia chebula Extract on Ischemic Stroke Rats	2018	Case-control	MCAO rats, serum		Arachidonic acid, L-Lactic acid, Phenylalanine	PC(16:0/20:4), Fumarate, L-Leucine, Succinic acid, L-Alanine, PC(20:4/20:4), LysoPC(18:0), LysoPC(16:0), L-Palmitoylcarnitine

Metabolite changes in the ipsilateral and contralateral cerebral hemispheres in rats with middle cerebral artery occlusion	2017	Case-control	MCAO rats, brain	1H-NMR	Lactate, Alanine, γ-Aminobutyric acid, Glycine	N-acetyl aspartate, Creatinine, Glutamate, Choline
Evolution of blood-brain barrier damage associated with changes in brain metabolites following acute ischemia	2015	Case-control	MCAO rats, brain	1H-MRS	Lactate, Alanine, γ-Aminobutyric acid, Glutamine, Myo-inositol	N-acetyl aspartate, N-acetylaspartylglutamate, Glutamate
Early metabolic changes following ischemia onset in rats: an in vivo diffusion-weighted imaging and 1H-magnetic resonance spectroscopy study at 7.0 T	2015	Case-control	MCAO rats, brain	1H-MRS	Lactate	N-acetyl aspartate
Analysis of serum metabolites for the discovery of amino acid biomarkers and the effect of galangin on cerebral ischemia	2013	Case-control	MCAO rats, serum	LC-MS/MS	Glutamic acid, Homocysteine, Methionine, Tryptophan, Alanine	N-acetyl aspartate
NMR analysis of the rat neurochemical changes induced by middle cerebral artery occlusion	2012	Case-control	MCAO rats, brain	1H-NMR	α-Glucose	Acetate, Glutamate, Glutamine, γ-Aminobutyric acid, Taurine, Aspartate, Malate, Creatine, Choline, Phosphorylcholine, *Myo*-inositol, uridine diphosphate/uridine triphosphate, Fumarate, Uracil, Xanthine, Inosine, Hypoxanthine, Nicotinurate
Dynamic Alterations of Brain Injury, Functional Recovery, and Metabolites Profile after Cerebral Ischemia/Reperfusion in Rats Contributes to Potential Biomarkers	2020		MCAO rats, brain	MALDI-MSI	D-2-Hydroxyglutaric acid, Petroselinic acid, Uracil, N-Acetyl-D-glucosamine, Uric acid, Isocitric acid, Docosahexaenoic acid, L-Isoleucine, L-Alloisoleucine, Pyruvic acid, Ratio of Pyruvic acid/L-Serine, Ratio of Oxoglutaric acid/L-Glutamic acid, D-Maltose, L-Pipecolic acid	Ratio of Citrulline/Ornithine, Ratio of Spermidine/Putrescine, Ratio of Uridine/Cytidine, Ratio of L-Glutamic acid/Oxoglutaric acid, L-Alanine, Beta-Glycerophosphoric acid, L-Homoserine, Niacinamide, Adenine, L-Arabitol, Ratio of Urea/L-Arginine

Metabolic basis of neuronal vulnerability to ischemia; an in vivo untargeted metabolomics approach	2020		MCAO rats, brain	LC-MS/MS	1-(beta-D-Ribofuranosyl)-1,4-dihydro nicotinamide(reduced nicotinamide riboside), Guanosine Diphosphate Mannose, Cytidine-5'-diphosphate, P1,P4-Bis(5'-xanthosyl) tetraphosphate, Biotinyl-5-AMP, N-omega-(ADP-D-ribosyl)-L-arginine (ADP-Ribosyl-L-arginine), 5-Aminoimidazole-4-carboxamide, Stachyose, Cytosine, Uridine diphosphate-N-acetylgalactosamine, UDP-N-Acetyl-D-hexosamine, Glutamylglutamic acid, Cytidine-5'-monophosphate-5-N-acety lneuraminic acid, Nicotinamide adenine dinucleotide, Phenylacetaldehyde, UDP-GlcNAc, Cytidine-5'-monophosphate-5-N-acety lneuraminic acid	Guanosine, γ-Glutamylglutamic acid, Xanthine, Adenine, Adenosine, Uracil, Cysteine, Acetyl-L-methionine, S-formylglutathione, γ-Glutamylglutamine, Inosine, Hypoxanthine, D-Ribose 5-phosphate, Glutathione, 4-Phosphopantothenoylcysteine, L-histidine, Cytidine, Aconitic acid, Arginine, Gluconic acid, Dopamine 4-O-sulfate, 6-Hydroxyhexanoic acid, 5-Hydroxyhexanoic acid, Uridine, Phosphohydroxypyruvic acid, N-acetyl-L-phenylalanine, D-mannitol, Acetyl Glutamic Acid, N-Formyl-L-glutamic acid, L-tryptophan, N-Acetyl-S-(N-methylcarbamoyl)cysteine, Lipoyl-amp, N-acetyl-L-leucine, N-acetylneuraminic acid, Nicotinic acid, Isocitric acid, Citric acid, L-phenylalanine, Cytidine, Diphosphate, Ethanolamine, Aspartic Acid, N-acetyl-l-aspartic acid, Glycerol 3-phosphate, N-acetyl-l-glutamic acid, Leucine, 5-amino-6-(5-phospho-D-ribitylamino)ura cil, N-Acetylornithine, Theanine, Phosphoethanolamine, Phosphodimethylethanolamine, N-acetylhexosamine 6-phosphate, N-Carbamoylsarcosine, D-hexose-1-phosphate, L-(-)-Threonine, Cystathionine, Citrulline, Serine, Pantothenol, Fumaric acid, Maleic acid, L-Pyroglutamic acid, L-Glutamic acid 5-phosphate, Homovanillic acid, Phenylpyruvic acid, Betalamic acid, Homocarnosine, D-Galactose, 2,5-Dichloro-4-oxohex-2-enedioate, Malic acid, 2-methylpropanedioic acid, Dopachrome, PE(40:6), Neuraminic acid,

						L-Xylonic acid, L-threonic acid, PE(38:6), succinic acid, D-4-Hydroxy-2-oxoglutarate
H/13C MR spectroscopic imaging of regionally specific metabolic alterations after experimental stroke	2008	Case-control	MCAO rats, brain	1H MRSI, MRI	Lactate	Glutamate, Glutamine

Detection of acute ischemic stroke and backtracking stroke onset time via machine learning analysis of metabolomics	2022	Case-control	MCAO rats, serum	LC-MS/MS	Glutamate, Isoleucine-alanine, Tryptophan	Trimethylamine n-oxide, Cis-9,10-epoxystearic acid, D-fructose
Metabolomic Profiling Reveals That Reprogramming of Cerebral Glucose Metabolism Is Involved in Ischemic Preconditioning-Induced Neuroprotection in a Rodent Model of Ischemic Stroke	2018	Case-control	MCAO rats, brain	GC-MS	Citrate<br>Glucose<br>Alanine<br>Lactate	Glutamate<br>N-Acetylaspartate
Neurochemical changes in unilateral cerebral hemisphere during the subacute stage of focal cerebral ischemia-reperfusion in rats: An ex vivo 1H magnetic resonance spectroscopy study	2018	Case-control	MCAO rats, brain	MRI	Glutamine, Myo-inositol, Lactate	N-acetyl aspartate, gamma-Aminobutyric acid, Glutamate, Succinate, Creatine
NMR metabolomic study of blood plasma in ischemic and ischemically preconditioned rats: an increased level of ketone bodies and decreased content of glycolytic products 24 h after global cerebral ischemia	2018	Case-control	MCAO rats, plasma	NMR	Acetone, Acetoacetate, beta-Hydroxybutyrate, Glucose	Lactate, Pyruvate, Acetate
Plasma low-molecular-weight thiol/disulphide homeostasis as an early indicator of global and focal cerebral ischemia	2017	Case-control	MCAO rats, plasma	HPLC	Glutathione	Homocysteine
Plasma low-molecular-weight thiol/disulphide homeostasis as an early indicator of global and focal cerebral ischemia	2017	Case-control	MCAO rats, brain	HPLC	Cysteine	
Untargeted metabonomic analysis of a cerebral stroke model in rats: a study based on UPLC-MS/MS	2023	Case-control	MCAO rats, fecal	UPLC-MS/MS	Niazicinin A, 5′-Methylthioadenosine	N-Methyl-1-deoxynojirimycin, Isogenistein 7-glucoside, Creatinine
Integrated 16S rRNA Gene Sequencing and LC-MS Analysis Revealed the Interplay Between Gut Microbiota and Plasma Metabolites in Rats With Ischemic Stroke.	2021	Case-control	MCAO rats, plasma	LC-MS/MS	Valine, Glutamine, Choline, Leucine, Acetylcholine, Methionine, Histidine, Phenylalanine, Tryptophan, 1-Stearoyl-2-arachidonoyl-sn-glycerol	
Combined Metabolipidomic and Machine Learning Approach in a Rat Model of Stroke Reveals a Deleterious Impact of Brain Injury on Heart Metabolism	2023	Case-control	MCAO rats, plasma	LC-MS/MS	Sphingomyelins (C16:0), Sphingomyelins (C14:1), Ceramides (d18:1/16:0), Hexosylceramides (d18:1/16:0), Hexosylceramides (d18:1/20:0), Trans-4-hydroxyproline, Lysine	Citrulline, Ornithine, 3-indolepropionic acid, Cholic acid, Desoxycholic acid, Phosphatidylcholines (aa C42:2), Phosphatidylcholines (aa C42:5), Phosphatidylcholines (aa C40:2), Phosphatidylcholines (ae C38:2), lyso-phosphatidylcholines (a C26:1), lyso-phosphatidylcholines(a C28:1)

		Case-control	MCAO rats, heart		Trans-4-hydroxyproline, Lysine, Serine, Asparagine	Acid 5-aminovaleric, Cholic acid, Betaine
Acrolein is involved in ischemic stroke-induced neurotoxicity through spermidine/spermine-N1-acetyltransferase activation	2020	Case-control study	Human urine; MCAO rats, plasma	HPLC-MS		3-hydroxypropyl mercapturic acid
Lipid peroxidation dysregulation in ischemic stroke: plasma 4-HNE as a potential biomarker?	2012	Case-control study	MCAO rats, plasma	ELISA	4-hydroxynonenal	
Targeted Lipid Profiling Discovers Plasma Biomarkers of Acute Brain Injury	2015	Case-control study	MCAO rats, plasma	HPLC-MS	SM 40:3, SM 36:1, SM 35:1, SM 38:2,<br>SM 38:1, SM 39:1, SM 36:2, SM 36:3	
