# Supplementary material for: Advances in metabolomics of biomarkers for ischemic stroke: From bench to clinic
Source: Neural Regen Res. 2025 Jul 5;21(8):3588–97. doi: 10.4103/NRR.NRR-D-24-01384 (PMC13452661; doi:10.4103/NRR.NRR-D-24-01384)
Supplement: Supplementary file 2 [file NRR-21-3588_Suppl2.pdf]

Additional Table 3 Summary of metabolomics studies in ischemic stroke patients

AUC info.	Title	Published year	Study design	Group	Specimens	Analytical platform	Up-regulated metabolites	Down-regulated metabolites
No related metabolite AUC value was offered	Plasma concentrations of C-reactive protein and total homocysteine in relation to the severity and risk factors for cerebrovascular disease	2007	Case-control study	IS patients with CVD (50) patients, healthy controls (20)	Plasma		Homocysteine	
No related metabolite AUC value was offered	Asymmetric dimethylarginine independently predicts fatal and nonfatal myocardial infarction and stroke in women: 24-year follow-up of the population study of women in Gothenburg	2008	Case-control study	IS patients (880, women)	Serum	HPLC	ADMA	
No related metabolite AUC value was offered	High plasma glutamate concentrations are associated with infarct growth in acute ischemic stroke	2008	Case-control study	IS patients (197)	Plasma		Glutamate	
AUC 0.68 (95 % CI, 0.55 ~ 0.8)	Oxidative stress and matrix metalloproteinase-9 in acute ischemic stroke: the Biomarker Evaluation for Antioxidant Therapies in Stroke (BEAT-Stroke) study	2008	Case-control study	IS patients (52), healthy controls (27)	Plasma	GC-MS	F2-isoprostanes	
No related metabolite AUC value was offered	Nonfasting triglycerides and risk of ischemic stroke in the general population	2008	Prospective study and Cross-sectional study	IS patients (13956)	Plasma		TG	

No related metabolite AUC value was offered	Targeted Metabolomic Evaluation of Arginine Methylation and Cardiovascular Risks: Potential Mechanisms Beyond Nitric Oxide Synthase Inhibition	2009	Prospective study	IS patients (1011)	Plasma	stable isotope dilution HPLC with on-line electrospray ionization tandem MS	ADMA, Symmetric dimethylarginine	
No related metabolite AUC value was offered	Diminished Global Arginine Bioavailability and Increased Arginine Catabolism as Metabolic Profile of Increased Cardiovascular Risk	2009	Prospective study	IS patients (1010)	Plasma	LC-MS/MS	Citrulline	
No related metabolite AUC value was offered	Metabolome-wide association study identifies multiple biomarkers that discriminate north and south Chinese populations at differing risks of cardiovascular disease: INTERMAP study	2010	Case-control study	IS patients (523 northern and 244 southern Chinese)	Urine	NMR	Dimethylglycine, Alanine, Lactate, Isoleucine, Leucine, Valine, Uromodulin, N-acetyl neuraminic acid, Pentanoic acid, Heptanoic acid, Methylguanidine	Hippurate, 4-Cresyl sulfate, Phenylacetylglutamine, 2-Pydroxyisobutyrate, Succinate, Creatine, Scyllo-inositol, Prolinebetaine, Transaconitate
No related metabolite AUC value was offered	Albuminuria and the risk of incident stroke and stroke types in older adults	2010	Longitudinal cohort study	IS patients (3287)	Urine		Cystatin C	
No related metabolite AUC value was offered	Possible Anandamide and Palmitoylethanolamide involvement in human stroke	2010	Case-control study	IS patients (10), healthy controls (8)	Blood	LC-MS/MS	Anandamide, Palmitoylethanolamide	
No related metabolite AUC value was offered	Triglycerides and risk of hemorrhagic stroke vs. ischemic vascular events: The Three-City Study	2010	Prospective cohort study	IS patients (8393)	Serum		TG	
No related metabolite AUC value was offered	Low serum homoarginine is a novel risk factor for fatal strokes in patients undergoing coronary angiography	2011	Case-control study	IS patients (3305)	Serum			L-Homoarginine

No related metabolite AUC value was offered	Asymmetric dimethylarginine as a risk marker for early-onset ischemic stroke in Indian population	2011	Case-control study	IS patients (201)	Plasma	SPE, HPLC	ADMA, Homocysteine	
No related metabolite AUC value was offered	1H-NMR-Based Metabolomics Study of Cerebral Infarction	2011	Case-control study	IS patients (81), healthy controls (74)	Plasma	1H-NMR	Formate, Glycolate, Lactate, Pyruvate	Glutamine, Lipid CH2CH2CC, Methanol, Valine, VLDL/LDL CH3
					Urine			Citrate, Creatinine, Dimethylamine, Glycine, Hippurate
No related metabolite AUC value was offered	Changes in plasma concentration of taurine in stroke	2011	Case-control study	IS patients (60), healthy controls (54)	Plasma	HPLC	Taurine	
No related metabolite AUC value was offered	A metabonomic approach applied to predict patients with cerebral infarction	2011	Case-control study	IS patients (67), healthy controls (62)	Serum	UPLC-TOF MS	Cysteine, S-Adenosyl-homocysteine, Oxidized glutathione, Hydroxyeicosatetraenoic acid, Hydroxyoctadecadienoic acid	Folic acid, Tetrahydrofolate, Adenosine, Aldosterone, Deoxocathasterone, Sucrose 6-phosphate, Betanin
No related metabolite AUC value was offered	Serum level and prognostic value of neopterin in patients after ischemic stroke	2012	Case-control study	IS patients (157)	Serum		Neopterin	
No related metabolite AUC value was offered	C-reactive protein and nitric oxide levels in ischemic stroke and its subtypes: correlation with clinical outcome	2012	Case-control study	IS patients (581), healthy controls (575)	Serum		NO	
No related metabolite AUC value was offered	Multiple biomarkers and risk of clinical and subclinical vascular brain injury: the Framingham Offspring Study	2012	Case-control study	IS patients (1901)	Plasma	HPLC	Homocysteine	

No related metabolite AUC value was offered	Lipid peroxidation dysregulation in ischemic stroke: plasma 4-HNE as a potential biomarker?	2012	Case-control study	IS patients (24), healthy controls (36)	Plasma	ELISA	4-hydroxynonenal	
No related metabolite AUC value was offered	Serum pentosidine, an advanced glycation end product, indicates poor outcomes after acute ischemic stroke	2012	Case-control study	IS patients (83)	Serum	ELISA	Pentosidine	
No related metabolite AUC value was offered	The Relation of Urinary 8-OHdG, A Marker of Oxidative Stress to DNA, and Clinical Outcomes for Ischemic Stroke	2012	Case-control study	IS patients (44)	Urine	ELISA	8-OHdG	
No related metabolite AUC value was offered	Homocysteine and C-reactive protein associated with progression and prognosis of intracranial branch atheromatous disease	2013	Retrospective study	BAD patients (179), LD patients (129)	Blood	Solid phase competitive chemiluminescent enzyme immunoassay	Homocysteine	
No related metabolite AUC value was offered	The effects of lipoprotein(a) and homocysteine on prognosis and risk factors in acute ischemic stroke.	2013	Case-control study	IS patients (41), healthy controls (33)	Plasma	HPLC	Homocysteine	
No related metabolite AUC value was offered	Biomarkers and functional outcomes from ischaemic cerebral events in women: a prospective cohort study	2013	Prospective cohort study	IS patients (27728)	Blood	Immunoturbidimetric assay, enzyme-linked immunosorbent assay, enzymatic assay, rate-blanked method that is based on the Jaffé reaction	Cholesterol, TG	
No related metabolite AUC value was offered	Biochemical and inflammatory biomarkers in ischemic stroke: translational study between humans and two experimental rat models	2014	Multi-center, prospective case-control study	IS patients (51), acute non-neurological diseases patients controls (17)	Plasma	HPLC	Glutamate	

No related metabolite AUC value was offered	The reliability and predictive ability of a biomarker of oxidative DNA damage on functional outcomes after stroke rehabilitation	2014	Case-control study	IS patients (61)	Urine	LC-MS/MS	8-OHdG	
No related metabolite AUC value was offered	Prognostic value of choline and betaine depends on intestinal microbiota-generated metabolite trimethylamine-N-oxide.	2014	Case-control study	IS patients (3903)	Plasma	LC-MS/MS	Choline, Betanin	
No related metabolite AUC value was offered	In older men, higher plasma testosterone or dihydrotestosterone is an independent predictor for reduced incidence of stroke but not myocardial infarction	2014	Case-control study	IS patients (3690, men)	Plasma	LC-MS/MS		Dihydrotestosterone
No related metabolite AUC value was offered	Fructosamine and Glycated Albumin and the Risk of Cardiovascular Outcomes and Death	2015	Prospective cohort study	IS patients (11104)	Serum		Fructosamine, Glycated Albumin	
No related metabolite AUC value was offered	Metabolomics predicts stroke recurrence after transient ischemic attack	2015	Case-control study	IS patients (293)	Plasma	LC-MS/MS	LysoPC (22:6)	LysoPC (16:0)
No related metabolite AUC value was offered	Targeted Lipid Profiling Discovers Plasma Biomarkers of Acute Brain Injury	2015	Case-control study	IS patients (14)	Plasma	HPLC-MS	SL:SM (36:0), Cer (42:1)	
No related metabolite AUC value was offered	Is serum total bilirubin useful to differentiate cardioembolic stroke from other stroke subtypes?	2015	Case-control study	IS patients (628)	Serum	MRI	Total bilirubin	

AUC 0.67 (95 % CI, 0.60 ~ 0.73)	Urinary biomarkers of oxidative and nitrosative stress and the risk for incident stroke: a nested case-control study from a community-based cohort	2015	Case-control study	IS patients (131)	Urine	LC-MS/MS	8-iso-PGF2α	
No related metabolite AUC value was offered	Highly Prevalent Hyperuricaemia is Associated with Adverse Clinical Outcomes Among Ghanaian Stroke Patients: An Observational Prospective Study	2015	Prospective observational study	IS patients (147)	Serum		Uric acid	
No related metabolite AUC value was offered	Serum malondialdehyde levels in patients with malignant middle cerebral artery infarction are associated with mortality.	2015	Observational, prospective and multicenter study	IS patients (50), healthy controls (100)	Serum		Malondialdehyde	
No related metabolite AUC value was offered	A metabonomic investigation on the biochemical perturbation in post-stroke patients with depressive disorder (PSD)	2016	Case-control study	IS patients (55), healthy controls (32)	Plasma	GC-MS	Aspartic acid	Cholesterol, Creatinine, Isoleucine, Linoleic acid, Oleic acid, Palmitic acid, Phenylalanine, Proline, Pyroglutamate, Serine, Stearic acid, Valine
No related metabolite AUC value was offered	Rapid and Sensitive Differentiating Ischemic and Hemorrhagic Strokes by Dried Blood Spot Based Direct Injection Mass Spectrometry Metabolomics Analysis	2016	Case-control study	IS patients (129), healthy controls (98)	Dried blood spot	HPLC-MS	Alanine, Citrulline, 3-hydroxyisovalerylcarnitine (C5–OH), C5–OH/C0, C5–OH/C8	

No related metabolite AUC value was offered	Metabolites of glutamate metabolism are associated with incident cardiovascular events in the PREDIMED PREvencion con DIeta MEDiterranea (PREDIMED) Trial	2016	Case-control study	IS patients (115), healthy controls (751)	Plasma	LC-MS/MS	Glutamate	Glutamine-to-glutamate ratio
No related metabolite AUC value was offered	Plasma branched-chain amino acids and incident cardiovascular disease in the PREDIMED trial	2016	Case-control study	IS patients (117), healthy controls (744)	Plasma	LC-MS/MS	Isoleucine, Leucine, Valine	
No related metabolite AUC value was offered	Plasma acylcarnitines and risk of cardiovascular disease: effect of Mediterranean diet interventions	2016	Case-control study	IS patients (188), healthy controls (751)	Plasma	LC-MS/MS	C2carnitine, C3DCCH3carnitine, C3carnitine, C4carnitine, C4OHcarnitine, C5carnitine, C5:1carnitine, C5:DCcarnitine, C6carnitine, C7carnitine, C8carnitine, C9carnitine, C10carnitine, C10:2carnitine, C12carnitine, C12:1carnitine, C14carnitine, C14:1carnitine, C14:2carnitine, C16carnitine, C18carnitine, C18:1carnitine, C18:1OHcarnitine, C18:2carnitine, C20carnitine, C20:4carnitine, C26carnitine	
No related metabolite AUC value was offered	Prognostic Significance of Blood Urea Nitrogen in Acute Ischemic Stroke	2017	Case-control study	IS patients (3355)	Blood		Urea nitrogen	

Functional outcome: Serum FFA level AUC 0.79 (95 % CI, 0.72–0.86), CSF FFA level AUC 0.81 (95 % CI, 0.72 ~ 0.87).<br>Mortality: Serum FFA level AUC 0.86 (95 % CI, 0.78–0.94), CSF FFA level AUC 0.87 (95 % CI, 0.81 ~ 0.94)	Elevated Serum and Cerebrospinal Fluid Free Fatty Acid Levels Are Associated with Unfavorable Functional Outcome in Subjects with Acute Ischemic Stroke	2017	Case-control study	IS patients (252)	Serum, Cerebrospinal fluid		FFA	
Alanine AUC 0.683<br>Citrate AUC 0.734<br>Glycine AUC 0.751<br>Isoleucine AUC 0.655<br>Lactate AUC 0.748<br>Leucine AUC 0.544<br>Serine AUC 0.871<br>Tyrosine AUC 0.758<br>Trytophan AUC 0.550<br>Methionine AUC 0.730<br>Erythronic acid AUC 0.747<br>Lysine AUC 0.714<br>Urea AUC 0.734<br>Purine AUC 0.646<br>Glutamate AUC 0.605<br>Proline AUC 0.583<br>PHE/TYR AUC 0.952	GC-MS-based metabolomics identifies an amino acid signature of acute ischemic stroke	2017	Case-control study	IS patients (40), healthy controls (29)	Serum	GC-MS	Lactate, Carbonate, Glutamate, PHE/TYR	Alanine, Citrate, Glycine, Isoleucine, Leucine, serine, Tyrosine, Methionine, Tryptophan, Erythronic acid, Urea, Purine, Hypoxanthine, Proline

Serine AUC 0.823<br>Isoleucine AUC 0.832<br>Betaine AUC 0.723<br>PC(5:0/5:0) AUC 0.927<br>LysoPE(18:2) AUC 0.785	Discovery of Metabolite Biomarkers for Acute Ischemic Stroke Progression.	2017	Case-control study	IS patients (26), healthy controls (23)	Serum	LC-MS/MS	Mannose<br>Galactose<br>Betaine<br>Carnitine<br>Acetylcarnitine<br>L-isoleucyl-L-proline<br>LysoPE(18:2)<br>LysoPE(20:2)<br>LysoPE(20:4)<br>LysoPE(20:5)	Alanine<br>Glycine<br>Serine<br>Isoleucine<br>Threonine<br>Aspartate<br>Proline Ornithine Lysine<br>Tricarballylic acid<br>Trihydroxy palmitic acid<br>LysoPC(16:0)<br>PA(18:3/0:0) PC(1:0/16:0)<br>PC(5:0/5:0) PI(22:2/0:0)
Uric acid AUC 0.778<br>LysoPE(0:0/18:3) AUC 0.730<br>LysoPC(18:2) AUC 0.748<br>Bilirubin AUC 0.859<br>Sphinganine AUC 0.754<br>Linoelaidyl carnitine AUC 0.903<br>LysoPC(16:0) AUC 0.794<br>Adrenoyl ethanolamide AUC 0.814<br>PE(15:0/22:1) AUC 0.903<br>PS(14:1/22:6) AUC 0.894<br>PC(14:0/20:4) AUC 0.787<br>PC(16:0/22:6) AUC 0.732	Potential serum biomarkers and metabonomic profiling of serum in ischemic stroke patients using UPLC/Q-TOF MS/MS	2017	Case-control study	IS patients (30), healthy controls (30)	Serum	UPLC/Q-TOF MS/MS	Uric acid, Sphinganine, Adrenal ethanolamide, Linoelaidyl carnitine, PE(15:0/22:1)	LysoPE(0:0/18:3), LysoPC(18:2), Bilirubin, PC(14:0/20:4), PS(14:1/22:6), LysoPC(16:0), PC(16:0/22:6)
no related metabolite AUC value was offered	Plasma ceramides, mediterranean diet, and incident cardiovascular disease in the predimed trial (prevención con dieta mediterránea)	2017	Case-control study	IS patients (62), healthy controls (348)	Serum	UPLC-QTOF-MS	Cer (16:0), Cer (22:0), Cer (24:0), Cer (24:1)	

Homocysteine sulfinic acid AUC 0.651<br>Cadaverine AUC 0.711<br>L-valine AUC 0.771<br>Nicotinamide AUC 0.653<br>N6-acetyl-l-lysine AUC 0.651<br>S-(2-methylpropionyl)-dihydrolipoamide-E AUC 0.713<br>Ubiquinone AUC 0.677<br>2-Oxoglutarate AUC 0.716<br>5-Aminopentanoate AUC 0.771	A metabolomic study on high-risk stroke patients determines low levels of serum lysine metabolites: a retrospective cohort study	2017	Retrospective study	IS patients (66), healthy controls (63)	Serum	LC-MS/MS	Homocysteine sulfinic acid	Cadaverine, L-lysine, L-valine, Nicotinamide, N6-acetyl-l-lysine, *S*-(2-methylpropionyl)-dihydrolipoamide-E, Ubiquinone, 2-Oxoglutarate, 5-Aminopentanoate
LysoPC (20:5) AUC 0.785<br>FFA (16:1) AUC 0.784<br>LysoPC (20:4) AUC 0.784<br>DG (38:6) AUC 0.754<br>TG (54:5) AUC 0.748<br>GluCer (38:2) AUC 0.745<br>TG (52:5) AUC 0.739<br>TG (54:4) AUC 0.728<br>LysoPC (24:0) AUC 0.722<br>TG (54:3) AUC 0.720<br>PE (35:2) AUC 0.703<br>TG (56:5) AUC 0.703<br>LysoPC (22:6) AUC 0.701	Lipidomic analysis of plasma in patients with lacunar infarction using normal-phase/reversed-phase two-dimensional liquid chromatography–quadrupole time-of-flight mass spectrometry	2017	Case-control study	IS patients (35), healthy controls (21)	Plasma	LC-QTOF-MS	DG (38:6), LysoPC (20:4), LysoPC (20:5), LysoPC (22:6), LysoPC (24:0), TG (52:5), TG (54:3), TG (54:4), TG (54:5), TG (56:5)	FFA (16:1), GluCer (38:2), PE (35:2)

Docosatrienoic acid AUC 0.809<br>Phytosphingosine AUC 0.769<br>Sphinganine AUC 0.837<br>Tetradecanedioic acid AUC 0.756<br>Glutamine AUC 0.776<br>LysoPE (0:0/22:0) AUC 0.707<br>Pyroglutamic acid AUC 0.753<br>2-Ketobutyric acid AUC 0.732	Metabolomics analysis on patients with ischemic stroke based on ultra-performance liquid chromatography with quadrupole-time-of-flight mass spectrometry	2017	Case-contro l study	IS patients (30), healthy controls (17)	Plasma	UPLC-QTOF-MS	Docosatrienoic acid, Phytosphingosine, Sphinganine, Tetradecanedioic acid	Glutamine, LysoPE (0:0/22:0), Pyroglutamic acid, 2-Ketobutyric acid
No related metabolite AUC value was offered	Plasma Homocysteine and Prognosis of Acute Ischemic Stroke: a Gender-Specific Analysis From CATIS Randomized Clinical Trial	2017	Case-contro l study	IS patients (3309)	Plasma		Homocysteine	
8-OHdG AUC 0.700 (95% CI, 0.626 ~ 0.773), MDA AUC 0.793 (95% CI, 0.731 ~ 0.856)	High Serum Levels of Malondialdehyde and 8-OHdG are both Associated with Early Cognitive Impairment in Patients with Acute Ischaemic Stroke	2017	Case-contro l study	IS patients (183)	Serum		Malondialdehyde, 8-OHdG	
No related metabolite AUC value was offered	Practical Application of Biogenic Amine Profiles for the Diagnosis of Patients with Ischemic Stroke	2018	Case-contro l study	IS patients (9), healthy controls (10)	Urine	MEKC method		Dopamine, L-tyrosine, 1-tryptophan, Noradrenaline

No related metabolite AUC value was offered	Metabolic Profile for Prediction of Ischemic Stroke in Chinese Hypertensive Population	2019	Case-control study	IS patients (66), healthy controls (66)	Plasma	UHPLC-MS/MS	(2S)-OMPT 2-Methyl-1-Pyrroline DL-Dihydrosphingosine Bufexamic Acid L-Thyroxine	9-cis-Retinal DL-Indole-3-Lactic Acid Methyl Ricinoleate D-Pipecolinic Acid 7,7-Dimethyl-5,8-Eicosadienoic Acid PC(15:0/0:0)[U] 10-Hydroxy-2-Decenoic Acid
No related metabolite AUC value was offered	Untargeted metabolomic analysis and pathway discovery in perinatal asphyxia and hypoxic-ischaemic encephalopathy	2019	Case-control study	HIE patients (30), healthy controls (30)	Serum	DI FT-ICR MS	Glycerol, L-Acetylcarnitine, Isobutyryl-L-carnitine, O-Butanoylcarnitine, Pseudouridine, Uridine, (+)-Catechin, (−)-Epicatechin, Cis-3,4-Leucopelargonidin, Uridine-triphosphate	2-Deoxyribonic acid, D-Ribose, D-Xylose, Melatonin
No related metabolite AUC value was offered	A prospective study of serum metabolites and risk of ischemic stroke	2019	Case-control study	IS patients (114), healthy controls (112)	Serum	GC-MS, LC-MS/MS	Tetradecanedioate, Hexadecanedioate	
No related metabolite AUC value was offered	A Multi-Cohort Metabolomics Analysis Discloses Sphingomyelin (32:1) Levels to be Inversely Related to Incident Ischemic Stroke	2019	Case-control study	IS patients (186), healthy controls (1643)	Serum	LC-MS/MS		Sphingomyelin (32:1)
No related metabolite AUC value was offered	Plasma alkylresorcinol metabolite, a biomarker of whole-grain wheat and rye intake, and risk of ischemic stroke: a case-control study	2019	Case-control study	IS patients (990), healthy controls (990)	Plasma	LC-MS/MS		3-(3,5-Dihydroxyphenyl)-1-propanoic acid

Day 1 Malondialdehyde AUC 0.77 (95% CI, 0.65 ~ 0.86), Day 4 Malondialdehyde AUC 0.82 (95% CI, 0.69 ~ 0.91), Day 8 Malondialdehyde AUC 0.84 (95% CI, 0.70 ~ 0.93)	Non-survivor patients with malignant middle cerebral artery infarction showed persistently high serum malondialdehyde levels	2019	Case-control study	Survivor MMCAI patients 34, non-survivor MMCAI patients control 34	Blood	Thiobarbituric acid-reactive substance (TBARS) method	Malondialdehyde	
No related metabolite AUC value was offered	Global metabolomics analysis of serum from humans at risk of thrombotic stroke	2020	Case-control study	IS patients (99), healthy controls (301)	Blood	LC-MS/MS	L-Tryptophan<br>Dopamine<br>3-Methoxytyramine<br>L-Methionine<br>S-Adenosyl-L-methionine<br>L-Leucine<br>L-Isoleucine<br>L-Cysteine<br>L-Homocysteine sulfinic acid<br>L-Proline<br>L-Arginine<br>2-Oxoglutarate<br>Guanosine<br>Linoleic acid<br>Cerebrosterol<br>Sphingosine<br>3-Ketosphingosine	N-acetylserotonin<br>Serotonin<br>Epinephrine<br>L-Phenylalanine<br>L-Valine<br>L-Hompcysteine<br>L-Cysteine sulfinic acid<br>L-Lysine<br>Carnitine<br>NAD<br>ATP<br>AMP<br>GTP<br>ITP<br>dATP<br>dAMP<br>N-acetylaspartate
No related metabolite AUC value was offered	Plasma levels of ceramides relate to ischemic stroke risk and clinical severity	2020	Case-control study	IS patients (202), controls (202)	Plasma	UPLC-MS/MS	Cer (d18:1/16:0), Cer (d18:1/22:0), Cer (d18:1/ 24:0)	
No related metabolite AUC value was offered	Role of serum uric acid in ischemic stroke: A case-control study in Bangladesh	2020	Case-control study	IS patients (169), healthy controls (169)	Blood	standard enzymatic method	Uric acid	

No related metabolite AUC value was offered	Integration of ultra-high-pressure liquid chromatography-tandem mass spectrometry with machine learning for identifying fatty acid metabolite biomarkers of ischemic stroke	2020	Case-control study	IS patients (197), healthy controls (122)	Plasma, brain	UHPLC-MS/MS	Arachidonic acid	9,10-DiHOME, 12,13-DiHOME, 9-HpODE, 9-HODE, 9-KODE, 13-HpODE, 13-HODE, 13-KODE, 8-iso-15-keto-PGF2∂, 14,15-DHET, 12-HETE, 11-HETE, Anandamide
No related metabolite AUC value was offered	Lipoxins, RevD1 and 9, 13 HODE as the most important derivatives after an early incident of ischemic stroke	2020	Case-control study	IS patients (75), healthy controls (35)	Blood	HPLC, GC	C16:0 palmitic acid, C16:1 palmitoleic acid, C18:1n9 ct oleic acid, C18:1 vaccinic acid, C18:3n6 gamma linoleic acid, C20:0 arachidic acid, C22:0 behenic acid, C22:1cis13, C24:0 lignoceric acid, C24:1 nervonic acid, Prostaglandin E2, 18RS HEPE, 15S HETE	C13:0 tridecanoic acid, C15:1 cis-10-pentadecanoid, C17:0 heptadecanoic acid, C17:1 cis-10-heptadecanoid acid, C18:0 stearic acid, C18:2n6c linoleic acid, C18:2n6t linoleic acid, C18:3n3 linolenic acid, C20:2 cis-11-eicodienoic acid, C20:3n3 cis-11-eicosatrienoic acid, C20:5n3 EPA, C22:2 cis-docodienoic acid, C22:4n6 docosatetraenoic acid, C22:5w3 docosapentaenoic acid, C22:6n3 DHA, LTX A4 5S(6R) 15R, 16RS HETE, 13S HODE, 9S HODE, 17RS HDHA, 5 oxo ETE, 5 HETE and RevD1
No related metabolite AUC value was offered	Acrolein is involved in ischemic stroke-induced neurotoxicity through spermidine/spermine-N1-acetyltransferase activation	2020	Case-control study	IS patients (49), healthy controls (99)	Human urine; MCAO rats, plasma	HPLC-MS		3-Hydroxypropyl mercapturic acid
No related metabolite AUC value was offered	Metabolomic profiling of fatty acid biomarkers for intracerebral hemorrhage stroke	2020	Case-control study	IS patients (65), healthy controls (70)	Serum	UHPLC-MS	17,18-Dihydroxy-eicosatetraenoic acid, 16-Hydroxy-docosahexaenoic acid, 15-Hydroxy-eicosatetraenoic acid	

S1P AUC 0.83 (95% CI, 0.74 ~ 0.92)	Serum Sphingosine 1-Phosphate (S1P): A Novel Diagnostic Biomarker in Early Acute Ischemic Stroke	2020	Case-control study	IS patients (72), healthy controls (65)	Serum	ELISA		S1P
No related metabolite AUC value was offered	Development of an ELISA for Measurement of Urinary 3-Hydroxypropyl Mercapturic Acid (3-HPMA), the Marker of Stroke	2020	Case-control study	IS patients (45), healthy controls (51)	Urine	ELISA		3-Hydroxypropyl mercapturic acid
No related metabolite AUC value was offered	Circulating Metabolites Differentiate Acute Ischemic Stroke from Stroke Mimics	2021	Case-control study	IS patients (508), healthy controls (102), stroke mimics (349)	Blood	UPLC-MS/MS	Palmitoylcarnitine, 3-Hydroxylbutyrylcarnitine	Proline, Tyrosine, Propionylcarnitine, Valine, Tryptophan, Leucine, Adenosine

SA1P AUC 0.719 (95% CI, 0.582 ~ 0.834), S1P AUC 0.701 (95% CI, 0.567 ~ 0.819)	Association of Plasma Metabolic Biomarker Sphingosine-1-Phosphate With Cerebral Collateral Circulation in Acute Ischemic Stroke	2021	Case-control study	IS patients (119), healthy controls (57)	Plasma	LC-MS/MS	Presqualene diphosphate<br>(4Z,7Z,10Z,13Z,16Z,19Z)-Docosahexaenoic-3-enoate<br>4-(3-Hydroxy-2-naphthyl)-2-oxobut-3-enoic acid<br>Phenylacetylglutamine<br>Hypoxanthine<br>L-Carnitine<br>p-Aminobenzoic acid<br>Trigonelline<br>N-Acetylputrescine<br>2-Amino-3-carboxymuconate semialdehyde<br>Aflatoxin B2<br>L-Leucine<br>2-Aminophenol<br>1,2-Bis(4-hydroxyphenyl)-2-propanol<br>Dephospho-CoA<br>(Indol-3-yl) acetamide<br>Cortisol<br>Salicin 6-phosphate<br>Stearidonic acid<br>Catechol<br>Androsta-1,4-diene-3,17-dione<br>Ajmaline<br>20-COOH-Leukotriene B4<br>cis-1,2-Dihydro-3-ethylcatechol<br>Prostaglandin H2<br>2-Fluorocyclohexadiene-cis-1,2-diol-1-carboxylate<br>11beta,21-Dihydroxy-5beta-pregnane-3,20-dione<br>3alpha,21-Dihydroxy-5beta-pregnane-11,20-dione<br>Creatine<br>Xanthine<br>5'-Benzoylphosphoadenosine<br>(1E,3E)-4-Hydroxybuta-1,3-diene-1,2,4-tricarboxylate<br>(1E)-4-Oxobut-1-ene-1,2,4-tricarboxy	S1P<br>SA1P<br>9,10-DHOME<br>Salicyluric<br>Trans,trans-Farnesyl diphosphate<br>Inosine<br>L-Glutamine<br>Phenylacetylglutamine<br>Ornithine<br>3-Hydroxybutyric acid<br>L-Citrulline<br>Catechol<br>Hydroquinone<br>Pantetheine<br>3,4-Dihydroxybenzoate<br>Taurine<br>2-Hydroxy-3-phenylpropenoate<br>Indole<br>L-Aspartate<br>Anisole<br>Benzyl alcohol<br>Corticosterone<br>p-Cumate<br>Itaconic acid<br>Citraconic acid<br>trans-o-Hydroxybenzylidenepyruvate<br>Aniline<br>5-Hydroxyisourate<br>Glycochenodeoxycholate 7-sulfate<br>4-Methylumbelliferyl acetate<br>5-Hydroxy-N-formylkynurenine<br>Isocitrate<br>Formyl-5 hydroxykynurenamine<br>(9Z)-Hexadecenoic acid<br>5-Sulfosalicylate<br>UMP<br>Pyruvic acid<br>20-Hydroxyleukotriene E4<br>Linoleate<br>Vanillate

						late<br>CMP-N-glycoloylneuraminate<br>trans-Zeatin riboside<br>N-Formyl-L-glutamate<br>4,4'-Diapolycopene<br>L-2-Aminoadipate adenylate<br>1-(5'-Phosphoribosyl)-5-formamido-4-imidazolecarboxamide<br>6-Aminohexanoate<br>S-(4 Methylthiobutylthiohydroximoyl)-L-cysteine<br>Isopentenyladenosine-5'-diphosphate<br>O-Acetylcarnitine<br>(15S)-15-Hydroxy-5,8,11-cis-13-trans-eicosatetraenoate<br>2-Hydroxyadipate<br>(2R,3S)-2,3-Dimethylmalate<br>Xylose<br>L-Xylulose<br>Caffeine<br>ADP-5-ethyl-4-methylthiazole-2-carboxylate<br>Paraoxon<br>21-Hydroxypregnenolone<br>7alpha Hydroxypregnenolone<br>Riboflavin<br>trans-Zeatin riboside diphosphate<br>Glycocholate<br>Theophylline<br>1,7-Dimethylxanthine<br>Pipecolic acid<br>L-Palmitoylcarnitine<br>Indolepyruvate<br>Benzene-1,2,4-triol<br>7-Dehydrodesmosterol<br>Progesterone<br>(4Z,7Z,10Z,13Z,16Z,19Z)-Docosahexaenoic acid	Cholesterol sulfate<br>Glycocholate<br>Ursodeoxycholic acid<br>Hyodeoxycholic acid<br>2-Phenylacetamide<br>(E)-Phenylacetaldoxime<br>N-Acetylanthranilate<br>Hippurate<br>5alpha Dihydrodeoxycorticosterone<br>Glyceric acid<br>Salicyluric acid

No related metabolite AUC value was offered	Serum metabolomic patterns in young patients with ischemic stroke: a case study	2021	Case-control study	IS patients (50), healthy controls (50)	Serum	LC-MS/MS	L-Methionine, Homocysteine, Glutamine, Uric acid, GCDCA, PE (18:0/20:4), PE (16:0/22:5)	L-Citrulline, Taurine, PC (16:2/22:6), PC (16:2/20:5), PC (15:0/18:2), and SM (d18:1/23:0), SM (d20:0/19:1), SM (d18:1/22:0), SM (d16:0/26:1), SM (d16:0/18:0), SM (d16:0/22:1), SM (d18:1/19:1), SM (d16:0/17:1), SM (d16:1/24:1), SM (d18:1/19:0)

12,13-DHOME AUC 0.820<br>9,10-DHOME AUC 0.820<br>Taurine AUC 0.821<br>trans,trans-Farnesyl diphosphate AUC 0.833<br>L-Glutamine AUC 0.782<br>4-(3-Hydroxy-2-naphthyl)-2-oxobut-3-enoic acid AUC 0.813<br>cis-4-(1'-Hydroxynaphth-2'-yl)-2-oxobut-3-enoate AUC 0.813<br>Inosine AUC 0.726<br>Indole AUC 0.803<br>2-Amino-3-carboxymuconate semialdehyde AUC 0.765<br>2-Hydroxy-3-phenylpropenoate AUC 0.727<br>Hypoxanthine AUC 0.686<br>L-2-Aminoadipate adenylate AUC 0.713<br>3-Dehydrosphinganine AUC 0.725<br>5'-Benzoylphosphoadenosine AUC 0.706<br>Dephospho-CoA AUC 0.771<br>(Indol-3-yl) acetamide AUC 0.738<br>Catechol AUC 0.710<br>Hydroquinone AUC 0.710<br>trans-Aconitic acid AUC 0.698<br>L-Xylulose AUC 0.702<br>Xylose AUC 0.702<br>Phenylacetylglutamine AUC 0.697<br>1,2-Bis(4-hydroxyphenyl)-2-propanol AUC 0.706<br>N-Acetylputrescine AUC	Phenylacetylglutamine, a Novel Biomarker in Acute Ischemic Stroke	2021	Case-control study	IS patients (751), healthy controls (200)	Plasma	LC-MS/MS	12,13-DHOME<br>9,10-DHOME<br>trans,trans-Farnesyl diphosphate<br>4-(3-Hydroxy-2-naphthyl)-2-oxobut-3-enoic acid<br>cis-4-(1'-Hydroxynaphth-2'-yl)-2-oxobut-3-enoate<br>Inosine<br>Indole<br>2-Amino-3-carboxymuconate semialdehyde<br>Hypoxanthine<br>L-2-Aminoadipate adenylate<br>5'-Benzoylphosphoadenosine<br>(Indol-3-yl) Acetamide<br>L-Xylulose<br>Xylose<br>Phenylacetylglutamine<br>1,2-Bis(4-hydroxyphenyl)-2-propanol<br>N-Acetylputrescine<br>20-COOH-Leukotriene B4<br>Anisole<br>Benzyl alcohol<br>Isopentenyladenosine-5'-diphosphate<br>trans-Zeatin riboside<br>Ajmaline<br>Phenylacetylglutamine<br>CMP-N-glycoloylneuraminate<br>11beta,21-Dihydroxy-5beta-pregnane-3,20-dione<br>3alpha,21-Dihydroxy-5beta-pregnane-11,20-dione<br>Cortisol<br>Methyl salicylate<br>L-Leucine<br>trans-o-Hydroxybenzylidenepyruvate<br>Salicyluric acid<br>3-Methyl-2-oxobutanoic acid<br>2-Fluorocyclohexadiene-cis,cis-1,2-diol-1-carboxylate	Taurine<br>L-Glutamine<br>2-Hydroxy-3-phenylpropenoate<br>3-Dehydrosphinganine<br>Dephospho-CoA<br>Catechol<br>Hydroquinone<br>trans-Aconitic acid<br>L-Citrulline<br>Androsta-1,4-diene-3,17-dione<br>Presqualene diphosphate<br>Ornithine<br>Pyruvic acid<br>Citraconic acid<br>Itaconic acid<br>13(S)-HPOT<br>9(S)-HPOT<br>Nicotine<br>Sphinganine 1-phosphate<br>3,4-Dihydroxybenzoate<br>Formyl-5-hydroxykynurenamine<br>Isocitrate<br>20-Hydroxyleukotriene E4<br>Guanine<br>Adenine<br>L-Glutamine<br>P1,P4-Bis(5'-guanosyl) tetraphosphate<br>5-Hydroxyisourate<br>N-(5-Phospho-D-ribosyl)anthranilate<br>2-Furoic acid<br>L-Malic acid

0.704<br>L-Citrulline AUC 0.698<br>20-COOH-Leukotriene B4 AUC 0.696<br>Androsta-1,4-diene-3,17-dione AUC 0.669<br>Presqualene diphosphate AUC 0.700<br>Ornithine AUC 0.692<br>Pyruvic acid AUC 0.686<br>Anisole AUC 0.708<br>Benzyl alcohol AUC 0.708<br>Isopentenyladenosine-5'-diphosphate AUC 0.701<br>trans-Zeatin riboside AUC 0.692<br>Ajmaline AUC 0.713<br>Phenylacetylglutamine AUC 0.700<br>CMP-N-glycoloylneuraminate AUC 0.690<br>11beta,21-Dihydroxy-5beta-pregnane-3,20-dione AUC 0.727<br>3alpha,21-Dihydroxy-5beta-pregnane-11,20-dione AUC 0.727<br>Citraconic acid AUC 0.692<br>Itaconic acid AUC 0.692<br>Cortisol AUC 0.704<br>Methyl salicylate AUC 0.700<br>13(S)-HPOT AUC 0.662<br>9(S)-HPOT AUC 0.662<br>Nicotine AUC 0.695<br>Sphinganine 1-phosphate AUC 0.661<br>3,4-Dihydroxybenzoate AUC 0.691<br>Formyl-5-hydroxykynurena							3-Hydroxybutyric acid<br>Aflatoxin B2<br>(9Z)-Hexadecenoic acid<br>ITP<br>ADP-5-ethyl-4-methylthiazole-2-carboxylate<br>Salicin 6-phosphate<br>4-Methylumbelliferyl acetate<br>5-Sulfosalicylate<br>Guanosine	

mine AUC 0.708<br>L-Leucine AUC 0.674<br>trans-o-Hydroxybenzylidenepyruvate AUC 0.714<br>Salicyluric acid AUC 0.688<br>Isocitrate AUC 0.673<br>3-Methyl-2-oxobutanoic acid AUC 0.669<br>20-Hydroxyleukotriene E4 AUC 0.657<br>2-Fluorocyclohexadiene-cis,cis-1,2-diol-1-carboxylate AUC 0.693<br>3-Hydroxybutyric acid AUC 0.686<br>Aflatoxin B2 AUC 0.692<br>Guanine AUC 0.757<br>Adenine AUC 0.677<br>L-Glutamine AUC 0.669<br>(9Z)-Hexadecenoic acid AUC 0.640<br>P1,P4-Bis(5'-guanosyl) tetraphosphate AUC 0.680<br>5-Hydroxyisourate AUC.696<br>ITP AUC 0.653<br>N-(5-Phospho-D-ribosyl)anthranilate AUC 0.669<br>ADP-5-ethyl-4-methylthiazole-2-carboxylate AUC 0.651<br>Salicin 6-phosphate AUC 0.607<br>2-Furoic acid AUC 0.657<br>4-Methylumbelliferyl acetate AUC 0.684<br>5-Sulfosalicylate AUC 0.663<br>L-Malic acid AUC 0.669<br>Guanosine AUC 0.685								

No related metabolite AUC value was offered	Predictive serum biomarkers of patients with cerebral infarction	2022	Case-control study	IS patients (28), healthy controls (27)	Serum	UPLC/Q-TOF MS/MS	5-Aminoimidazole-4-carboxamide, Pipecolic acid, L-Tyrosine, Tyramine, Vanylglycol, L-2,4-diaminobutyric acid, 2-Phenylglycine, 5,6-Epoxy-8,11,14-eicosatrienoic acid	Dimethyltryptamine, 1-Hexadecanoyl-sn-glycero-3-phosphoethanolamine, 1-Heptadecanoyl-sn-glycero-3-phosphochline, Linoleic acid, Palmitic acid
No related metabolite AUC value was offered	Plasma ceramides are associated with outcomes in acute ischemic stroke patients	2022	Case-control study	IS patients (87), healthy controls (30)	Plasma	UHPLC-ESI-MS/MS	Cer(d18:1/14:0), Cer(d18:1/16:0), Cer(d18:1/18:0)	S1P, Cer(d18:1/22:0), Cer(d18:1/24:0)
Argininosuccinic acid AUC 0.897 (95% CI), beta-D-glucosamine AUC 0.909 (95% CI), Glycerophosphocholine AUC 0.816 (95% CI), L-Abrine AUC 0.841 (95% CI), L-Pipecolic acid AUC 0.804 (95% CI)	Metabolomic Characterization of Acute Ischemic Stroke Facilitates Metabolomic Biomarker Discovery	2022	Case-control study	IS patients (22), healthy controls (22)	Serum	LC-MRM/MS		Argininosuccinic acid, beta-D-glucosamine, Glycerophosphocholine, L-abrine, L-pipecolic acid
Taurine AUC 0.861<br>Oleoylcarnitine AUC 0.845<br>Cer (34:2) AUC 0.931<br>LysoPC (16:0) AUC 0.901<br>22:6-OH/LysoPC AUC 0.889<br>TAG58:7-FA22:4 AUC 0.865<br>PE (22:6/P-18:0) AUC 0.931<br>GlcCer (d18:0/18:0) AUC 0.917<br>DG (44:0) AUC 0.909<br>Creatinine AUC 0.818	Integrated Metabolomics and Lipidomics Approach for the Study of Metabolic Network and Early Diagnosis in Cerebral Infarction	2022	Case-control study	CI patients (47), healthy controls (59)	Serum	UPLC-Q-TOF/MS	PE (22:6/P-18:0)<br>GlcCer (d18:0/18:0)<br>DG (44:0)<br>Creatinine	Taurine<br>Oleoylcarnitine<br>Cer (34:2)<br>LysoPC (16:0)<br>22:6-OH/LysoPC<br>TAG58:7-FA22:4

No related metabolite AUC value was offered	Integrating serum proteomics and metabolomics to compare the common and distinct features between acute aggressive ischemic stroke (APIS) and acute non-aggressive ischemic stroke (ANPIS)	2022	Case-control study	AIPS patients (24), ANPIS patients (53) healthy controls (29)	Serum	GC-MS	L-Cysteine<br>Hexadecanoic acid<br>Stearic acid	L-Alanine<br>Glycine<br>Urea<br>L-Leucine<br>L-isoleucine<br>L-Methionine<br>L-Proline<br>L-Serine<br>L-Threonine<br>L-Phenylalanine<br>L-Lysine
220 metabolites AUC 0.76 (95% CI, P = 0.006), 63 selected metabolites AUC 0.81 (95% CI, P < 0.001)	Untargeted metabolomics predicts the functional outcome of ischemic stroke	2021	Case-control study	IS patients (150)	Blood	Q-TOF MASS	Ascochitine<br>10-Acetoxytoxol<br>Dihydromethysticin<br>Lactucin<br>5-De-O-methyltoddanol<br>2-[2-hydroxy-3-(3-hydroxyphenyl)propyl]benzene-1,3,5-triol<br>7-hydroxy-6-(4-hydroxy-3-methylbut-2-en-1-yl)-8-methoxy-2H-chromen-2-one<br>6-[(3,3-dimethyloxiran-2-yl)methyl]-7-hydroxy-8-methoxy-2H-chromen-2-one<br>3-(7-methoxy-2-oxo-2H-chromen-8-yl)-2,2-dimethylpropanoic acid<br>2-[2-hydroxy-3-(4-hydroxyphenyl)propyl]benzene-1,3,5-triol<br>8-[(3,3-dimethyloxiran-2-yl)methyl]-6-hydroxy-7-methoxy-2H-chromen-2-one<br>8-{[3-(hydroxymethyl)-3-methyloxiran-2-yl]methyl}-7-methoxy-2H-chromen-2-one<br>6-hydroxy-8-(2-hydroxy-3-methylbut-3-en-1-yl)-7-methoxy-2H-chromen-2-one<br>8-(2,4-dihydroxy-3-methylidenebutyl)-7-methoxy-2H-chromen-2-one<br>8-(1,2-dihydroxy-3-methylbut-3-en-1-	2-(2-Methylbutanoyl)-9-(3-methyl-2E-pentenoyl)-2b,9a-dihydroxy-4Z,10(14)-oplopadien-3-one<br>8-Methyl-3-hentriacontene,12Z,15Z)/18:4(6Z,9Z,12Z,15Z))<br>Vinaginsenoside R12<br>Fasciculic acid C<br>LysoPC(18:0)<br>LysoPC(0:0/18:0)<br>2-acetyl-1-alkyl-sn-glycero-3-phosphocholine<br>Kaempferol 3-rhamnoside 7-galacturonide<br>6"-(4-Carboxy-3-hydroxy-3-methylbutanoyl)hyperin<br>6-(4-{3,5-dihydroxy-4-oxo-7-[(3,4,5-trihydroxy-6-methyloxan-2-yl)oxy]-4H-chromen-2-yl}phenoxy)-3,4,5-trihydroxyoxane-2-carboxylic acid<br>3,4,5-trihydroxy-6-{[5-hydroxy-2-(4-hydroxyphenyl)-4-oxo-7-[(3,4,5-trihydroxy-6-methyloxan-2-yl)oxy]-4H-chromen-3-yl]oxy}oxane-2-carboxylic acid<br>3,4,5-trihydroxy-6-{[3-hydroxy-2-(4-hydroxyphenyl)-4-oxo-7-[(3,4,5-trihydroxy-6-methyloxan-2-yl)oxy]-4H-chromen-5-yl]oxy}oxane-2-carboxylic acid

						yl)-7-methoxy-2H-chromen-2-one<br>8-[2-hydroxy-2-(2-methyloxiran-2-yl)ethyl]-7-methoxy-2H-chromen-2-one<br>7,8-dihydroxy-6-methoxy-3-(2-methylbut-3-en-2-yl)-2H-chromen-2-one<br>4-methoxy-6-[3-(4-methoxyphenyl)oxiran-2-yl]-5,6-dihydro-2H-pyran-2-one<br>Phenol sulphate<br>N-Acetylneuraminic acid<br>N-Acetyl-a-neuraminic acid<br>Tyrosyl-Glutamate<br>6-[(3-carboxy-2-methylprop-2-en-1-yl)oxy]-3,4,5-trihydroxyoxane-2-carboxylic acid<br>3,4,5-trihydroxy-6-[(4-hydroxy-3-methylbut-2-enoyl)oxy]oxane-2-carboxylic acid<br>6-(3,3-dimethyloxirane-2-carbonyloxy)-3,4,5-trihydroxyoxane-2-carboxylic acid<br>6-[(2Z)-2-carboxy-2-ethylideneethoxy]-3,4,5-trihydroxyoxane-2-carboxylic acid<br>3,4,5-trihydroxy-6-{[(2E)-2-(hydroxymethyl)but-2-enoyl]oxy}oxane-2-carboxylic acid<br>6-[(3-carboxy-3-methylprop-2-en-1-yl)oxy]-3,4,5-trihydroxyoxane-2-carboxylic acid<br>3,4,5-trihydroxy-6-[(4-hydroxy-2-methylbut-2-enoyl)oxy]oxane-2-carboxylic acid<br>6-(2,3-dimethyloxirane-2-carbonyloxy)-3,4,5-trihydroxyoxane-2-carboxylic acid<br>Deoxyuridine<br>Topaquinone<br>Pemirolast<br>Serylthreonine	3,4,5-trihydroxy-6-{[5-hydroxy-2-(4-hydroxyphenyl)-4-oxo-6-[3,4,5-trihydroxy-6-(hydroxymethyl)oxan-2-yl]-4H-chromen-7-yl]oxy}oxane-2-carboxylic acid<br>3,4,5-trihydroxy-6-{[7-hydroxy-2-(4-hydroxyphenyl)-4-oxo-6-[3,4,5-trihydroxy-6-(hydroxymethyl)oxan-2-yl]-4H-chromen-5-yl]oxy}oxane-2-carboxylic acid<br>6-(4-{5,7-dihydroxy-4-oxo-6-[3,4,5-trihydroxy-6-(hydroxymethyl)oxan-2-yl]-4H-chromen-2-yl}phenoxy)-3,4,5-trihydroxyoxane-2-carboxylic acid<br>6-(4-{5,7-dihydroxy-4-oxo-8-[3,4,5-trihydroxy-6-(hydroxymethyl)oxan-2-yl]-4H-chromen-2-yl}phenoxy)-3,4,5-trihydroxyoxane-2-carboxylic acid<br>3,4,5-trihydroxy-6-{[7-hydroxy-2-(4-hydroxyphenyl)-4-oxo-8-[3,4,5-trihydroxy-6-(hydroxymethyl)oxan-2-yl]-4H-chromen-5-yl]oxy}oxane-2-carboxylic acid<br>3,4,5-trihydroxy-6-{[5-hydroxy-2-(4-hydroxyphenyl)-4-oxo-8-[3,4,5-trihydroxy-6-(hydroxymethyl)oxan-2-yl]-4H-chromen-7-yl]oxy}oxane-2-carboxylic acid<br>6-({5,7-dihydroxy-4-oxo-2-phenyl-8-[3,4,5-trihydroxy-6-(hydroxymethyl)oxan-2-yl]-4H-chromen-6-yl}oxy)-3,4,5-trihydroxyoxane-2-carboxylic acid<br>6-({6,7-dihydroxy-4-oxo-2-phenyl-8-[3,4,5-trihydroxy-6-(hydroxymethyl)oxan-2-yl]-4H-chromen-5-yl}oxy)-3,4,5-trihydroxyoxane-2-carboxylic acid<br>6-({5,6-dihydroxy-4-oxo-2-phenyl-8-[3,4,5-trihydroxy-6-(hydroxymethyl)oxan-2-yl]-4H-chromen-7-yl}oxy)-3,4,5-trihydroxyoxane-2-carboxylic acid

						Threoninyl-Serine<br>Betalamic acid<br>2,4-Dihydroxy-7-methoxy-2H-1,4-benzoxazin-3(4H)-one<br>Gentisuric acid<br>2-{[(3,4-dihydroxyphenyl)(hydroxy)methylidene]amino}acetic acid<br>2-{[(2,4-dihydroxyphenyl)(hydroxy)methylidene]amino}acetic acid<br>2-{[(3,5-dihydroxyphenyl)(hydroxy)methylidene]amino}acetic acid<br>2-{[(2,3-dihydroxyphenyl)(hydroxy)methylidene]amino}acetic acid<br>Dibutyl disulfide<br>2-O-Methyl-L-fucose<br>1,8-Octanedithiol<br>Gluten exorphin A5<br>Nilvadipine<br>Dimethyl 2-galloylgalactarate<br>2,12-Tetradecadiene-4,6,8,10-tetrayne	6-(3-{5,7-dihydroxy-4-oxo-8-[3,4,5-trihydroxy-6-(hydroxymethyl)oxan-2-yl]-4H-chromen-2-yl}phenoxy)-3,4,5-trihydroxyoxane-2-carboxylic acid<br>3,4,5-trihydroxy-6-{[7-hydroxy-2-(3-hydroxyphenyl)-4-oxo-8-[3,4,5-trihydroxy-6-(hydroxymethyl)oxan-2-yl]-4H-chromen-5-yl]oxy}oxane-2-carboxylic acid<br>3,4,5-trihydroxy-6-{[5-hydroxy-2-(3-hydroxyphenyl)-4-oxo-8-[3,4,5-trihydroxy-6-(hydroxymethyl)oxan-2-yl]-4H-chromen-7-yl]oxy}oxane-2-carboxylic acid<br>3,4,5-trihydroxy-6-{[5-hydroxy-2-(4-hydroxyphenyl)-7-methoxy-4-oxo-8-(3,4,5-trihydroxyoxan-2-yl)-4H-chromen-6-yl]oxy}oxane-2-carboxylic acid<br>6-{4-[5,6-dihydroxy-7-methoxy-4-oxo-8-(3,4,5-trihydroxyoxan-2-yl)-4H-chromen-2-yl]phenoxy}-3,4,5-trihydroxyoxane-2-carboxylic acid<br>3,4,5-trihydroxy-6-{[6-hydroxy-2-(4-hydroxyphenyl)-7-methoxy-4-oxo-8-(3,4,5-trihydroxyoxan-2-yl)-4H-chromen-5-yl]oxy}oxane-2-carboxylic acid<br>3,4,5-trihydroxy-6-{2-hydroxy-5-[5-hydroxy-7-methoxy-4-oxo-8-(3,4,5-trihydroxyoxan-2-yl)-4H-chromen-2-yl]phenoxy}oxane-2-carboxylic acid<br>3,4,5-trihydroxy-6-{2-hydroxy-4-[5-hydroxy-7-methoxy-4-oxo-8-(3,4,5-trihydroxyoxan-2-yl)-4H-chromen-2-yl]phenoxy}oxane-2-carboxylic acid<br>6-{[2-(3,4-dihydroxyphenyl)-7-methoxy-4-oxo-8-(3,4,5-trihydroxyoxan-2-yl)-4H-chromen-5-yl]oxy}-3,4,5-trihydroxyoxane-2-carboxylic acid<br>3,4,5-trihydroxy-6-{[5-hydroxy-2-(3-hydroxyphenyl)-4-oxo-6-[3,4,5-trihyd

							roxy-6-(hydroxymethyl)oxan-2-yl]-4H-chromen-7-yl]oxy}oxane-2-carboxylic acid<br>3,4,5-trihydroxy-6-{[7-hydroxy-2-(3-hydroxyphenyl)-4-oxo-6-[3,4,5-trihydroxy-6-(hydroxymethyl)oxan-2-yl]-4H-chromen-5-yl]oxy}oxane-2-carboxylic acid<br>6-(3-{5,7-dihydroxy-4-oxo-6-[3,4,5-trihydroxy-6-(hydroxymethyl)oxan-2-yl]-4H-chromen-2-yl}phenoxy)-3,4,5-trihydroxyoxane-2-carboxylic acid<br>6-(4-{5,7-dihydroxy-4-oxo-3-[(3,4,5-trihydroxy-6-methyloxan-2-yl)oxy]-4H-chromen-2-yl}phenoxy)-3,4,5-trihydroxyoxane-2-carboxylic acid<br>3,4,5-trihydroxy-6-{[7-hydroxy-2-(4-hydroxyphenyl)-4-oxo-3-[(3,4,5-trihydroxy-6-methyloxan-2-yl)oxy]-4H-chromen-5-yl]oxy}oxane-2-carboxylic acid<br>8-Methyl-3-hentriacontene<br>Metanephrine<br>1-Methyl-6-phenyl-1H-imidazo[4,5-b]pyridin-2-amine-Ethoxy-3-methoxybenzaldehyde<br>2-Phenyl-1,3-dioxolane-4-methanol<br>1-(2,4-Dihydroxyphenyl)-1-butanone<br>Benzaldehyde glyceryl acetal<br>Propylparaben<br>Ethyl p-anisate<br>4-Methoxybenzyl acetate<br>Tenuazonic acid<br>4-Hydroxy-2,6,6-trimethyl-3-oxo-1,4-cyclohexadiene-1-carboxaldehyde<br>Propyl 2-furanacrylate<br>Desglymidodrine<br>3-(3-Hydroxyphenyl)-2-methylpropionic acid<br>L-Metanephrine

							10-hydroxy-(2E,8E)-decadien-4-ynoic Acid<br>1,2-octadienoylglycine<br>1,3-octadienoylglycine<br>1,4-octadienoylglycine<br>1,5-octadienoylglycine<br>1,6-octadienoylglycine<br>1,7-octadienoylglycine<br>2,3-octadienoylglycine<br>2,4-octadienoylglycine<br>2,5-octadienoylglycine<br>2,6-octadienoylglycine<br>2,7-octadienoylglycine<br>3,4-octadienoylglycine<br>3,5-octadienoylglycine<br>3,6-octadienoylglycine<br>3,7-octadienoylglycine<br>4,5-octadienoylglycine<br>4,6-octadienoylglycine<br>4,7-octadienoylglycine<br>5,6-octadienoylglycine<br>5,7-octadienoylglycine<br>6,7-octadienoylglycine<br>4-ethenyl-2,6-dimethoxyphenol<br>2-(4-methoxyphenyl)propanoic acid<br>3-(4-methoxyphenyl)propanoic acid<br>4-(3,4-dihydroxyphenyl)butan-2-one<br>3-(2-hydroxy-3-methoxyphenyl)propanal<br>2-(3-hydroxyprop-1-en-1-yl)-6-methoxyphenol<br>3-methoxy-5-(prop-2-en-1-yl)benzene-1,2-diol<br>2-methoxy-4-[(oxiran-2-yl)methyl]phenol<br>2-methoxy-4-(prop-2-en-1-yl)benzene-1,3-diol<br>4-methoxy-6-(prop-2-en-1-yl)benzene-1,3-diol<br>2-(3-hydroxyprop-1-en-1-yl)-5-metho

								xyphenol<br>[3-(4-methoxyphenyl)oxiran-2-yl]methanol<br>2-hydroxy-3-(4-methoxyphenyl)propanal<br>3-hydroxy-3-(4-methoxyphenyl)propanal<br>3-(2-hydroxyphenyl)butanoic acid<br>3-hydroxy-3-phenylbutanoic acid<br>3-(4-hydroxyphenyl)butanoic acid<br>4-hydroxy-3-phenylbutanoic acid<br>2-hydroxy-3-phenylbutanoic acid<br>3-hydroxy-4-(4-hydroxyphenyl)butan-2-one<br>4-(2,4-dihydroxyphenyl)butan-2-one<br>4-hydroxy-4-(4-hydroxyphenyl)butan-2-one<br>1-Methyl-6-phenyl-1H-imidazo[4,5-b]pyridin-2-amine<br>1-Methyl-6-phenyl-1H-imidazo[4,5-b]pyridin-2-amine-Ethoxy-3-methoxybenzaldehyde<br>8-Methyl-3-hentriacontene,12Z,15Z)/18:4(6Z,9Z,12Z,15Z))<br>3-Methoxybenzenepropanoic acid<br>Coniferyl alcohol<br>4-Ethoxy-3-methoxybenzaldehyde<br>Piritramide<br>PS (18:4(6Z,9Z,12Z,15Z)/18:4(6Z,9Z,12Z,15Z))
No related metabolite AUC value was offered	Targeting succinate metabolism to decrease brain injury upon mechanical thrombectomy treatment of ischemic stroke	2023	Case-control study	IS patients brain tissue (8)	Brain	LC-MS/MS	Succinate	

no related metabolite AUC value was offered	Assessing the causal effect of genetically predicted metabolites and metabolic pathways on stroke	2023	Case-control study	IS patients (34217), controls (412479)	Serum	MR analysis	Margaret (17:0), Aspartate	
AcCa(C2) AUC 0.839, AcCa(C12:1) AUC 0.820, AcCa(C14) AUC 0.673, AcCa(C18:1-OH) AUC 0.883	Lipidomic analysis identifies long-chain acylcarnitine as a target for ischemic stroke	2023	Case-control study	IS patients (41), healthy controls (33)	Blood	MS	AcCa(C2), AcCa(C12:1), AcCa(C14), AcCa(C18:1-OH)	
no related metabolite AUC value was offered	Plasma metabolomic profiling of patients with transient ischemic attack reveals positive role of neutrophils in ischemic tolerance	2023	Case-control study	IS patients (20), healthy controls (20)	Plasma	LC-MS/MS	All-trans 13,14 dihydroretinol, 20-Carboxyleukotriene B4, Prostaglandin B2, Cortisol, 9-KODE	
no related metabolite AUC value was offered	A Metabolomic Signature of Ischemic Stroke Showing Acute Oxidative and Energetic Stress	2023	Case-control study	IS patients (79), healthy controls (40)	Blood	LC-MS/MS	Methionine sulfoxide, Acetyl (C2) carnitine, Propionyl (C3) carnitine, Oleoyl (C18:1) carnitine, diacyl PC aa 40:2, triacylglyceride 16:0/37:3	Choline, Arachidonic acid, Docosahexaenoic acid, LysoPC 18:0, Acyl-alkyl PC ae 34:3

no related metabolite AUC value was offered	LC-MS/MS based metabolomic analysis of serum from patients with cerebrovascular stenosis	2023	Case-control study	IS patients (18), healthy controls (18)	Serum	UPLC-MS/MS	Deoxycholic acid, Cystine, Hydroquinidine, N-(3-Methoxybenzy)oleamide, Aurapten, Thymol-beta-D-glucoside, 5-hydroxy-2-(4-hydroxyphenyl)-4-oxo-3,4-dihydro-2H-chromen-7-yl 2-O-(6-deoxy-alpha-L-mannopyranosyl)-beta-D-glucopyranoside, Arginine, beta-Guanidinopropionic acid, Cholic acid, Clopidogrel carboxylic acid, 5,7,4'-Trihydroxy-8-methylflavanone, 1-(4-methoxyphenyl)ethanone	L-Tyrosine, L-5-Oxoproline, 1-(2-(1H-indol-3-yl)ethyl)urea, Gentiannine, Sweroside, 2-Aminophenol, Ajugol, Piperine, Inosine, Caffeine, Rosin, PFSA-Cl, Cotinine, Cyclamate, Glycocholate, Chlorothalonil-4-hydroxy
no related metabolite AUC value was offered	Discovery and validation of circulating stroke metabolites by NMR-based analyses using patients from the MISS and UK Biobank	2023	Case-control study	IS patients (260), healthy controls (160)	Serum	NMR	Phenylalanine, 3- hydroxybutyrate	Polyunsaturated fatty acids, PC, Phosphoglycerides
no related metabolite AUC value was offered	Searching for Metabolic Markers of Stroke in Human Plasma via NMR Analysis	2023	Case-control study	low stroke risk (LSR, control) 85, moderate stroke risk (MSR) 94, high stroke risk (HSR) 18	Plasma	NMR	Dimethyl sulfone, Asparagine, Formate, Creatinine, Isoleucine, Formate	
no related metabolite AUC value was offered	Lipidomic signature of stroke recurrence after transient ischemic attack	2023	Case-control study	Stroke recurrence patients (37), non-stroke recurrence controls (415)	Plasma	MS/MS		TG (56:1), TG (63:0), TG (58:2), TG (50:5), Phosphoethanolamine (P-18:0/18:2), DG (38:5), TG (53:7)

Glycocholic acid AUC 0.67	Identification of a potential prognostic plasma biomarker of acute ischemic stroke via untargeted LC-MS metabolomics	2023	Case-control study	IS patients (100)	Plasma	LC-MS/MS	LysoPC18:4, Verimol J	LysoPC 22:4, 9,10,13-TriHOME, Methyluric acid, Glycocholic acid, Palmitoyl glucuronide
Carnitine C10:1-OH AUC 0.946, Carnitine C10:1 AUC 0.870, Cer (d18:0/16:0) AUC 0.778	Identification of three potential novel biomarkers for early diagnosis of acute ischemic stroke via plasma lipidomics	2023	Case-control study	IS patients (30), healthy controls (30)	Blood	UPLC-MS/MS	PA (18:1_22:4)<br>PA (16:0_16:1)<br>CerP (d18:1/18:1)<br>CerP (d18:1/18:0)<br>Cer (d18:0/18:0)<br>PE (16:1_16:0)<br>Cer (d18:0/20:0)<br>PI (16:0_16:1)<br>LNAPE (16:1/N-16:0)<br>LPI (16:1)<br>PA(18:1_18:1)<br>Cer(d18:1/16:0)<br>PA(16:0_18:1)	PE (O-18:1_16:0)<br>PE (P-18:0_20:5)<br>PE (O-15:1_22:6)<br>PE (O-14:1_22:6)<br>PE (O-17:1_18:2)<br>PE (O-17:1_20:5)<br>PE (O-18:1_22:2)<br>Carnitine C8:1-OH<br>Carnitine C9:0<br>Carnitine C10:1-OH<br>Carnitine C10:1<br>PMeOH (18:0_18:2)<br>PE (O-18:1_20:3)<br>PE (O-18:1_22:6)<br>PE (O-15:1_20:4)<br>PE (O-17:1_20:4)<br>PE (O-16:1_20:4)<br>PE (O-18:1_20:4)<br>PE (O-17:2_20:4)<br>PE (20:4_22:2)<br>PE (O-19:1_20:4)<br>PE (O-16:0_18:2)
No related metabolite AUC value was offered	Investigating sphingolipids as biomarkers for the outcomes of acute ischemic stroke patients receiving endovascular treatment	2023	Case-control study	IS patients (75), healthy controls (19)	Plasma	UHPLC-ESI-MS/MS	Cer (d18:1/16:0), Cer (d18:1/18:0), Cer (d18:1/20:0), Cer (d18:1/24:1)	S1P

PC (40:3P)(rep) AUC 0.722<br>ChE (20:5) AUC 0.700<br>TG (18:0p/18:1/18:1) AUC 0.724	Plasma lipidomic profiling reveals six candidate biomarkers for the prediction of incident stroke in patients with hypertension	2024	Case-control study	hypertensive patients with newly developed stroke (30), patients with hypertension (30), patients at high risk of stroke (30), healthy controls (30)	Plasma	UHPLC-MS	PC (17:0/22:6)<br>PC (37:4)(rep)<br>PG (48:3)<br>PC (39:7)<br>PE (18:1/22:6)<br>ChE (20:5)<br>SM (d37:4)<br>PE (18:0/22:6)<br>PE (16:0/22:6)<br>PC (35:6)<br>SM (d35:3)<br>PC (40:5)(rep)<br>PC (39:7p)(rep)	SM (d41:7)<br>PC (40:3p)(rep)<br>TG (18:1/22:1/22:1)<br>MG (24:3)<br>TG (18:1/18:2/23:0)<br>PC (22:6/23:5)<br>TG (18:1/18:2/24:0)<br>TG (18:0p/18:1/18:1)<br>TG (18:1/18:2/24:1)<br>TG (18:0e/18:1/18:2)<br>Co (q9)<br>TG (22:0/18:2/18:2)<br>SiE (18:2)<br>TG (18:0p/16:0/18:2)<br>TG (16:0e/18:1/18:2)<br>PE (42:2p)(rep)<br>PC (39:2p)<br>TG (18:0p/18:0/18:1)<br>PC (33:3)<br>PS (39:4)<br>PC (16:1/20:4)<br>TG (24:1/18:2/18:2)<br>PC (36:5)<br>TG (18:1/18:2/21:0)<br>MG (29:5)<br>PC (40:2p)<br>ChE(18:3)(rep)<br>PC (16:0e/20:3)
No related metabolite AUC value was offered	Targeted Metabolomic Biomarkers for Stroke Subtyping	2024	Case-control study	IS patients (316), healthy controls (30)	Serum	LC-MS/MS	Lysine, LysoPC acyl 16:0, Serine, Threonine	Putrescine, Kynurenine

Discovery cohort:<br>CerP(d18:1/18:1) AUC 0.802<br>CerP(d18:1/20:3) AUC 0.804<br>CerP(d18:1/18:0) AUC 0.880<br>CerP(d18:1/16:0) AUC 0.825<br>SM(d18:1/26:1) AUC 0.830<br>Cer(d18:0/20:0) AUC 0.855<br>Validation cohort:<br>CerP(d18:1/20:3) AUC 0.631<br>CerP(d18:1/18:1) AUC 0.738<br>CerP(d18:1/18:0) AUC 0.680<br>CerP(d18:1/16:0) AUC 0.717<br>SM(d18:1/26:1) AUC 0.782<br>Cer(d18:0/20:0) AUC 0.807<br>Whole cohort:<br>CerP(d18:1/20:3) AUC 0.717<br>CerP(d18:1/18:1) AUC 0.760<br>CerP(d18:1/18:0) AUC 0.766<br>CerP(d18:1/16:0) AUC 0.762<br>SM(d18:1/26:1) AUC 0.804<br>Cer(d 18:0/20.0) AUC 0.827	The identification of novel stroke-related sphingolipid biomarkers using UPLC-MS/MS	2024	Case-contro l study	IS patients (56), healthy controls (56)	Plasma	UPLC-MS/MS	Cer(d18:1/24:1)<br>Cer(d18:1/18:1)<br>Cer(d18:1/20:1)<br>Cer(d18:1/22:1)<br>Cer(d18:1/26:1)<br>Cer(d18:0/24:1)<br>Cer(d18:0/24:0)<br>Cer(d18:1/24:0(2OH))<br>Cer(d18:0/16:0)<br>Cer(d18:0/18:0)<br>Cer(d18:0/20:0)<br>CerP(d18:1/20:3)<br>CerP(d18:1/18:1)<br>CerP(d18:1/20:0)<br>CerP(d18:1/18:0)<br>CerP(d18:1/16:0)<br>Cer(t18:0/20:0(2OH))<br>Cer(t18:0/24:0(2OH))<br>Cer(t18:0/26:0)<br>SHexCer(d18:1:/24:1)<br>SHexCer(d18:1:/24:0)<br>SHexCer(d18:1:/16:0)<br>SHexCer(d18:1:/16:0(OH))<br>HexCer(d18:2/24:1)<br>HexCer(d18:1/18:1)<br>HexCer(d18:1/20:1)<br>HexCer(d18:1/24:1)<br>SM(d18:1/25:1)<br>SM(d18:1/26:1)<br>SM(d18:0/18:0)	SPH(d20:1)

8-iso-PGF2α: 8-Iso-prostaglandin F2α; 8-OHdG: 8-hydroxy-2’-deoxyguanosine; ADMA: asymmetric dimethylarginine; Cer: ceramide; CerP: ceramide 1-phosphate; Co: coenzyme; DG: diglyceride; FFA: free fatty acid; GluCer: glucosylceramide; LysoPC: lysophosphatidylcholine; LysoPE: lysophosphatidylethanolamine; MDA: malondialdehyde; MG: monoglycerde; PA: phosphatidic acid; PC: phosphatidylcholine; PE: phosphatidylethanolamine; PHE/TYR: the ratio of phenylalanine to tyrosine; S1P: sphingosine 1-phosphate; SA1P: sphinganine-1-phosphate; SiE: sitosteryl ester; SL: sphingolipid; SM: sphingomyelin; TB: total bilirubin; TG: triglycerides.
